# Mitochondrial protein E2F3d, a distinctive E2F3 product, mediates hypoxia-induced mitophagy in cancer cells

**DOI:** 10.1038/s42003-018-0246-9

**Published:** 2019-01-03

**Authors:** Keigo Araki, Keiko Kawauchi, Wataru Sugimoto, Daisuke Tsuda, Hiroya Oda, Ryosuke Yoshida, Kiyoshi Ohtani

**Affiliations:** 1 Department of Biomedical Chemistry, School of Science and Technology, Kwansei Gakuin University, 2-1 Gakuen, Sanda, Hyogo 669-1337 Japan; 2 Frontiers of Innovative Research in Science and Technology, Konan University, 7-1-20 Minatojima-Minamimachi, Chuo-ku, Kobe, Hyogo 650-0047 Japan

## Abstract

Mitochondrial damage is caused by changes in the micro-environmental conditions during tumor progression. Cancer cells require mechanisms for mitochondrial quality control during this process; however, how mitochondrial integrity is maintained is unclear. Here we show that E2F3d, a previously unidentified E2F3 isoform, mediates hypoxia-induced mitophagy in cancer cells. Aberrant activity and expression of the E2F3 transcription factor is frequently observed in many cancer cells. Loss of retinoblastoma (Rb) protein family function increases the expression of E2F3d and E2F3a. E2F3d localizes to the outer mitochondrial membrane and its cytosolic domain contains an LC3-interacting region motif. Overexpression of E2F3d induces mitochondrial fragmentation and mitophagy, suggesting that E2F3d plays an important role in mitophagy. Furthermore, depletion of E2F3s attenuates hypoxia-induced mitophagy and increases intracellular levels of reactive oxygen species, which is reversed by the reintroduction of E2F3d. This study presents another key player that regulates mitochondrial quality control in cancer cells.

## Abstract (web summary)

Keigo Araki and colleagues show that E2F3d, a previously unidentified isoform of transcription factor E2F3, mediates hypoxia-induced mitophagy in cancer cells. This study discovers another mitophagy receptor that regulates mitochondrial quality control.

## Introduction

Mitochondria are double-membrane-bound organelles that produce most of the energy required for cellular activity through oxidative phosphorylation^1,2^. In addition, they have an important role in the regulation of cell death and signaling^3^. Mitochondrial dysfunction is strongly linked to pathological phenotypes, and mitochondrial quality control is therefore critical for proper cell function. Genetic alterations in tumor suppressor genes and oncogenes (such as *TP53* and *RAS*), which frequently underlie tumorigenesis, impair mitochondrial integrity^4,5^ and changes in the micro-environmental conditions during tumor progression (such as hypoxia and nutrient deprivation) cause mitochondrial damage^6^. Thus, mitochondrial quality control is thought to be required for tumor progression.

Mitophagy is an important mechanism for mitochondrial quality control^7–9^. Damaged mitochondria are specifically recognized and sequestered into autophagosomes by isolation membranes. These are then fused with lysosomes, allowing catabolism of the cargo. This selective type of autophagy is triggered either by the PINK1–Parkin pathway or by mitophagy receptors^8,9^. In mammalian cells, mitophagy receptors localize to the outer mitochondrial membrane (OMM) and contain an LC3-interacting region (LIR) motif in their cytosolic domain^10^. LC3 family proteins are structural proteins of the autophagosomal membrane, and the interaction between mitophagy receptors and LC3 through the LIR motif contributes to recognition and subsequent sequestration of damaged mitochondria by autophagosomes^10^. Clearance of dysfunctional mitochondria by mitophagy opposes tumorigenesis during cancer initiation but contributes to cell growth and survival during tumor progression^3^. Mitophagy is therefore considered important for tumors’ progression from the benign to the malignant stage, but the precise mechanisms by which mitophagy is regulated require further research.

E2F transcription factors are key regulators of cell cycle progression^11^. They bind to DNA as heterodimers with dimerization partner proteins and control the expression of cellular genes encoding cell cycle regulators. E2Fs can be divided into two subgroups based on their transcriptional properties, namely activator E2Fs and repressor E2Fs^11,12^. E2F1–3a function as transcriptional activators, and their expression is aberrantly induced by loss of retinoblastoma (Rb) protein family function, which is intimately associated with deregulated proliferation of many cancer cells^13^. Two E2F3 isoforms, E2F3a and E2F3b, have distinct expression patterns using alternative promoters at the *E2F3* locus, where *E2F3b* is transcribed from an intronic promoter within the first intron of *E2F3a*^14^. The transcripts of *E2F3a* and *E2F3b* overlap extensively and share most exons (though their initial exons differ) making the E2F3b protein nearly identical to the E2F3a protein, except that it lacks the N-terminal domain of E2F3a^11,12^. E2F3a is particularly important for promoting cell proliferation, and *E2F3* gene amplification and overexpression of E2F3 are detected in several types of human cancers, especially high-grade cancers^12,15^. Herein, we reveal that E2F3d, a distinctive E2F3 product, localizes to the OMM and does not possess the canonical functional properties of the E2F family of transcription factors. E2F3d physically interacts with LC3 and overexpression of E2F3d induces mitochondrial fragmentation and mitophagy, indicating that E2F3d functions as a mitophagy receptor in mammalian cells. Our study provides new insights into mechanisms for the acquisition of mitophagic capacity in cancer cells.

## Results

### E1A induces the expression of unrecognized E2F3 isoforms

**Fig. 1:**
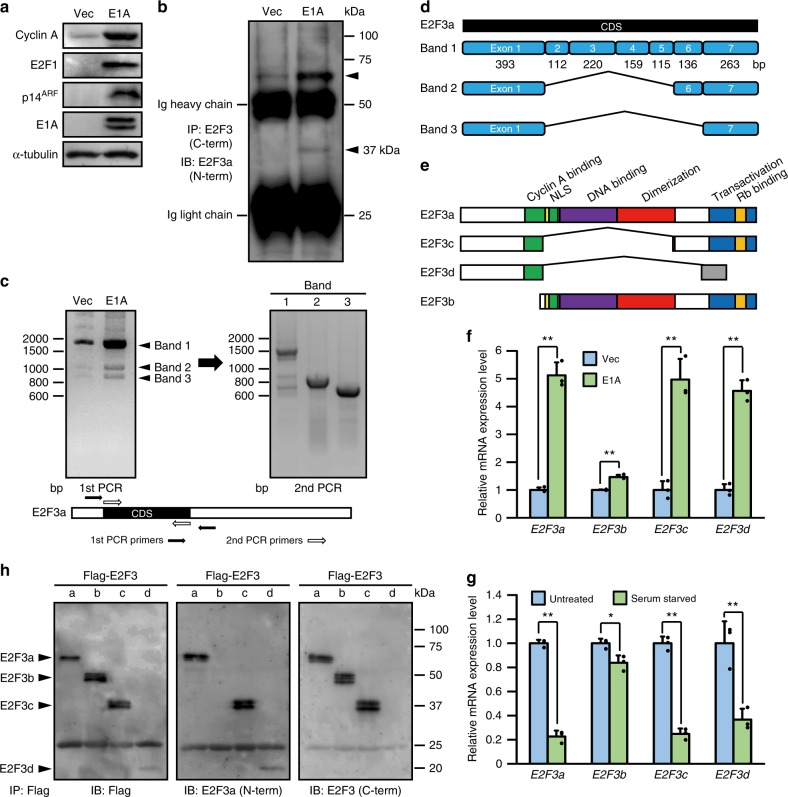
E2F3c and E2F3d are newly identified E2F3 products. **a**–**f** HFFs were infected with control (Vec) or E1A-expressing retroviruses. **a** Cell lysates were analyzed by immunoblotting to examine the protein levels of E2F target genes. **b** Cell lysates were immunoprecipitated with an antibody against the C-terminal domain of human E2F3 proteins and probed with an antibody against the N-terminal domain of human E2F3a protein. **c** Total mRNAs were subjected to RT-PCR. The RT-PCR products observed in E1A-expressing cells (bands 1–3) were extracted separately and further amplified in the second reaction using inner primers nested within the first primers. **d** mRNA structure of newly identified *E2F3* members. Schematic diagrams represent the exon composition of the RT-PCR products. **e** Domain structure of E2F3 members. **f** cDNA samples were subjected to qRT-PCR using primer sets specific for each *E2F3* member. Values shown represent the means of three independent experiments. Error bars represent SD. **P < 0.01. **g** cDNA samples from asynchronously growing (untreated) and quiescent (serum starved) HFFs were subjected to qRT-PCR as described in **f**. Values shown represent the means of three independent experiments. Error bars represent SD. *P < 0.05 and **P < 0.01. **h** 293 T cells were transfected with expression vectors encoding Flag-tagged individual E2F3 members. Cell lysates were subjected to immunoprecipitation with anti-Flag antibody-agarose beads followed by immunoblotting with antibodies directed against Flag or the N- or C-terminal domain of human E2F3a protein. Full-size scans of immunoblots are shown in Supplementary Information

Loss of Rb protein family function leads to aberrant expression of E2F target genes, including activator E2Fs, which influences various phenotypes of many human cancers^12,13^. Indeed, when adenovirus E1A, a potent viral oncoprotein that acts by inactivating the Rb protein family, was retrovirally introduced into human foreskin fibroblasts (HFFs), expression of E2F target genes (e.g., cyclin A, E2F1, and p14^ARF^) was induced (Fig. 1a). To date, E2F3 is known to have two isoforms, E2F3a and E2F3b; *E2F3a*, but not *E2F3b*, is an E2F target gene^14^. To specifically examine the expression of E2F3a, E2F3 proteins were immunoprecipitated from cell lysates using an antibody against the E2F3 C-terminal domain and were then probed with an antibody directed against the E2F3a-specific N-terminal domain. Notably, in E1A-expressing cells, two bands with increased intensity were detected at around 57 kDa, the expected size of E2F3a, and around 37 kDa (Fig. 1b). We then sought to identify the cDNA coding for this unexpected protein, which contained the N- and C-terminal peptide sequences of E2F3a. Reverse transcription-PCR (RT-PCR) using primers surrounding the start and end of the E2F3a coding sequence (CDS) showed that three pronounced products (bands 1–3) were detected in E1A-expressing cells, while the largest product alone was detectable in control cells (Fig. 1c). The complete CDS of E2F3a is composed of seven exons (exons 1–7). The DNA sequence of band 1 corresponded to the full CDS of E2F3a. The DNA sequence of band 2 corresponded to exons 1, 6, and 7 of the E2F3a CDS, and that of band 3 corresponded to exons 1 and 7 (Fig. 1d). We refer to these sequences as *E2F3c* and *E2F3d*, respectively. Domain structural analysis demonstrated that the E2F3c and E2F3d proteins lack a nuclear localization signal, DNA-binding domain, and dimerization domain. E2F3c contains a conserved C-terminal domain that mediates transcriptional activation and association with Rb protein family, but the C-terminal domain of E2F3d is not identical to that of E2F3a because of a frameshift (Fig. 1e). Quantitative RT-PCR (qRT-PCR) showed that E1A expression increased the transcription of *E2F3a*, *E2F3c*, and *E2F3d* to a similar extent (Fig. 1f). *E2F3a*, but not *E2F3b*, is transcribed in a cell cycle-dependent manner^14^ and the mRNAs of *E2F3c* and *E2F3d*, as well as *E2F3a*, were present at low levels in quiescent cells (Fig. 1g). These results imply that *E2F3c* and *E2F3d* are splice variants of *E2F3a*, and that their transcription is regulated in the same manner as *E2F3a*. Immunoblotting for individual E2F3 proteins verified that E2F3a, E2F3c, and E2F3d contain identical N-terminal domains and that E2F3a, E2F3b, and E2F3c contain identical C-terminal domains (Fig. 1h). Taken together, these results suggest that the unexpected protein observed in Fig. 1b was E2F3c.

### E2F3d is an integral OMM protein

**Fig. 2:**
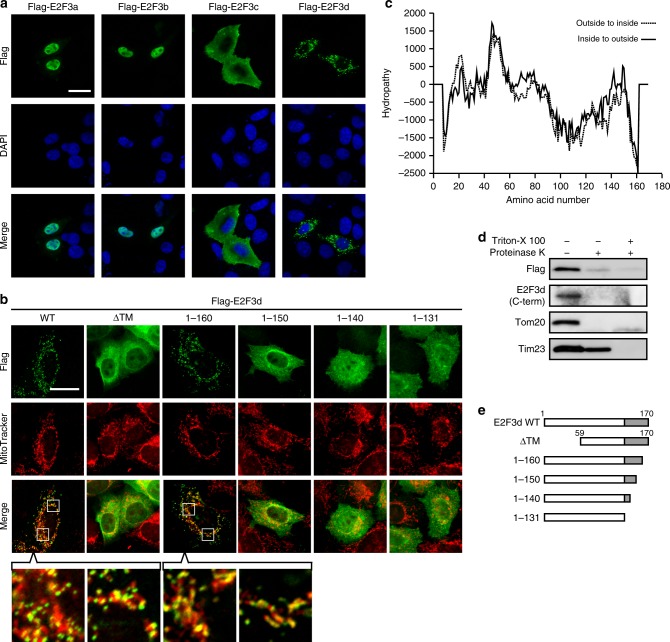
E2F3d localizes to the OMM. **a** HeLa cells were transfected with expression vectors encoding Flag-tagged individual E2F3 members and then immunostained with anti-Flag antibody. Scale bar, 20 μm. **b** HeLa cells were transfected with Flag-tagged WT or deletion mutant E2F3d constructs and incubated with MitoTracker Red CMXRos (200 nM) for 30 min. Cells were then immunostained with anti-Flag antibody. The boxed areas are shown at higher magnification in the lowest panel. Scale bar, 20 μm. **c** Prediction of TM domains for E2F3d. A hydropathy plot was created using the TMpred algorithm. **d** Mitochondria were isolated from HeLa cells transfected with a Flag-tagged WT E2F3d construct and treated with proteinase K in the absence or presence of Triton X-100, followed by immunoblotting. **e** Schematic diagrams of full-length E2F3d (WT) and a series of its deletion mutants. Unique C-terminal domains of E2F3d are highlighted in gray. Full-size scans of immunoblots are shown in Supplementary Information. Please see Supplementary Fig. 1

Localization of E2F3a and E2F3b was confined to the nucleus (Fig. 2a). E2F3c was distributed throughout the cytoplasm, whereas E2F3d also localized to the cytoplasm but exhibited a diverse aggregated appearance (Fig. 2a). We next dissected the subcellular localization of E2F3d and found that it localized to the mitochondria (Fig. 2b). To elucidate whether E2F3d is a mitochondrial membrane protein, we first employed a computational approach to predict the transmembrane (TM) protein topology. The hydropathy profile of E2F3d predicted two TM domains (amino acids 12–31 and 37–58) with high hydrophobicity in the N-terminal region of E2F3d (Fig. 2c). Proteinase treatment of isolated mitochondria, in which E2F3d protein tagged with a Flag epitope at its N-terminus was expressed, demonstrated that the N- and C-terminal domains of E2F3d and the OMM protein, Tom20, were digested by proteinase treatment, but a considerable amount of the inner mitochondrial membrane protein, Tim23, remained after treatment (Fig. 2d). On the basis of these results, we propose that E2F3d is an integral OMM protein with two TM domains, and that the N- and C-terminal domains of E2F3d are oriented toward the cytosol. We investigated whether E2F3d is anchored in the OMM via its predicted TM domains and found that the E2F3d protein lacking the putative TM domains (ΔTM) was distributed in the cytoplasm (Fig. 2b, e).

Translocation of most mitochondrial proteins is dependent on a conventional mitochondrial targeting sequence located at their N-termini^16^. However, given that E2F3c contains an identical N-terminal domain to E2F3d and did not localize to the mitochondria (Fig. 2a), mitochondrial targeting of E2F3d is thought to have been achieved by its unique C-terminal domain, not the N-terminal domain. We then generated a series of C-terminal deletion mutants (Fig. 2e) and assessed their subcellular localization. While we observed mitochondrial localization of E2F3d 1–160 (amino acids 1–160), E2F3d 1–150 was dispersed in the cytoplasm (Fig. 2b). Mitochondrial translocation of proteins without an N-terminal mitochondrial targeting sequence is mediated by interaction with cytosolic chaperones, such as Hsp70 (ref. ^16^). To explore the interaction between E2F3d and Hsp70, we expressed C-terminal deletion mutants of E2F3d in the cytoplasm by truncation of the TM domains and analyzed using a bimolecular fluorescence complementation (BiFC) technique. E2F3d 59–160 successfully generated a BiFC signal with Hsp70 in the cytoplasm, but E2F3d 59–150 failed (Supplementary Fig. 1). These results suggest that the N- and C-terminal domains of E2F3d contribute to its mitochondrial anchorage and translocation, respectively, and both are essential for the mitochondrial localization of E2F3d.

### Mitophagy is triggered by E2F3d

**Fig. 3:**
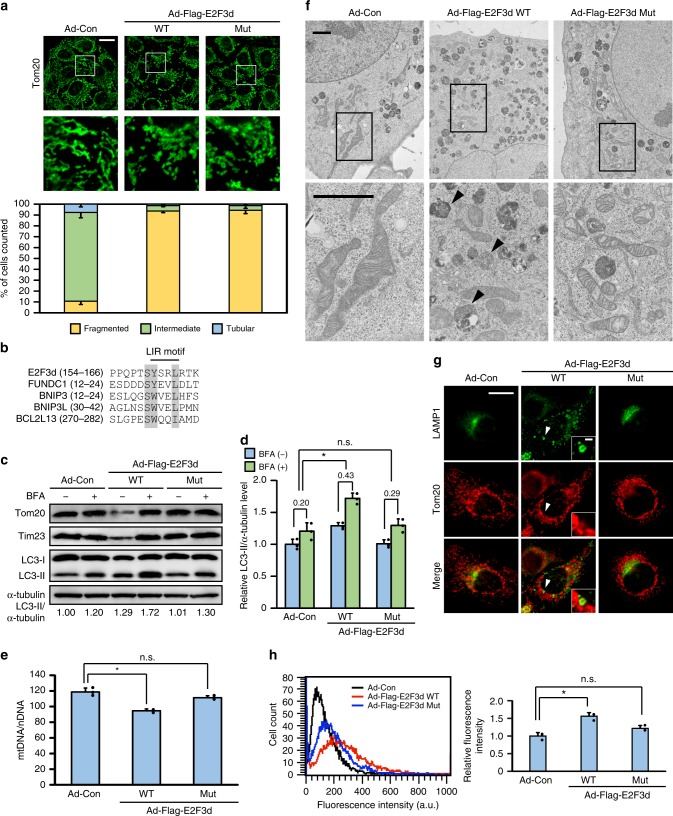
E2F3d induces mitochondrial fragmentation and mitophagy. HeLa cells were infected with control adenovirus (Ad-Con) or adenoviruses expressing Flag-tagged WT or LIR mutant E2F3d (Ad-Flag-E2F3d WT or Mut). **a** Cells were immunostained with anti-Tom20 antibody. The bottom images show magnifications of the boxed areas in the top images. Scale bar, 20 μm. The graph shows the percentage of cells exhibiting fragmented, intermediate, or tubular mitochondrial morphologies. Data are presented as the mean of three independent experiments (≥100 cells). Error bars represent SD. **b** Sequence alignment of the putative LIR motif in E2F3d and other known mitophagy receptors. The shaded regions indicate highly conserved residues. **c** Cells were treated with BFA (100 nM) for 16 h and cell lysates were examined by immunoblotting. LC3-II protein levels were normalized to α-tubulin to determine the differences in levels between the absence and presence of BFA. **d** Statistical analysis of quantified LC3-II protein levels in **c**. Data are presented as the mean of three independent experiments. Error bars represent SD. *P < 0.05. n.s. not significant. **e** Mitochondrial DNA (mtDNA) content was normalized to nuclear DNA (nDNA). Data are presented as the mean of three independent experiments. Error bars represent SD. *P < 0.05. n.s. not significant. **f** Cells were analyzed by electron microscopy. The boxed areas are shown at higher magnification. Arrowheads represent mitochondrion-like structures surrounded by a limiting membrane. Scale bars, 2 μm. **g** Cells were immunostained with anti-LAMP1 and anti-Tom20 antibodies. Scale bar, 20 μm. The areas pointed by arrowheads are shown at higher magnification. Scale bar, 2 μm. **h** The uptake of mitochondria by lysosomes was evaluated by mitophagy assay. Data are presented as the mean of three independent experiments (≥10,000 cells). Error bars represent SD. *P < 0.05. n.s. not significant, a.u. arbitrary units. Full-size scans of immunoblots are shown in Supplementary Information. Please see Supplementary Figs. 2–4

To examine the initial responses of mitochondria to E2F3d, we adenovirally expressed E2F3d and analyzed the mitochondrial morphology. Expression of E2F3d promoted a dramatic morphological change, with more than 90% of cells exhibiting fragmented mitochondria compared with approximately 10% of control cells (Fig. 3a). Mitochondrial fission in mammalian cells is primarily mediated by dynamin-related protein 1 (Drp1), a large GTPase^1,2^. We then examined whether Drp1 was indispensable for E2F3d-induced mitochondrial fragmentation. HeLa cells in which Drp1 was knocked down by shRNA exhibited elongated mitochondria. Expression of E2F3d induced mitochondrial fragmentation in the Drp1 knockdown cells, although to a slightly lesser extent than in control cells (Supplementary Fig. 2a, b). In addition, these findings were recapitulated in cells expressing a dominant-negative Drp1 mutant, in which its enzymatic activity was disrupted by a lysine to alanine substitution (K38A) in the GTPase domain (Supplementary Fig. 2c, d). These results suggest that Drp1 is not essential for E2F3d-induced mitochondrial fragmentation.

Previous studies have demonstrated that the introduction of mitophagy receptors induces mitochondrial fragmentation^17–19^ and that Drp1 is dispensable for mitochondrial fragmentation during mitophagy^20^. We next searched for the LIR motif ([W/F/Y]xx[L/I/V]), a pivotal feature of mammalian mitophagy receptors, in E2F3d and found that it contains a YxxL motif at its C-terminus (Fig. 3b), which is exposed to the cytosol. In addition, a serine residue flanking the aromatic residues in the LIR motif, which is involved in the interaction with LC3 (ref. ^10^), is also conserved in E2F3d. In vitro pull-down assays verified the physical interaction between purified E2F3d protein and glutathione *S*-transferase (GST)-LC3B fusion protein (Supplementary Fig. 3a). An E2F3d mutant protein containing tyrosine/leucine to alanine substitutions in the LIR motif (Y160A and L163A) displayed distinctly reduced interaction with LC3B protein (Supplementary Fig. 3a). Moreover, E2F3d interacted with LC3A, GABARAP, and GABARAPL2 (Supplementary Fig. 3b). These are other human homologs of ATG8, which plays an essential role in autophagosome biogenesis and the recruitment of autophagy cargo into autophagosomes^21^. In wild-type (WT) E2F3d-expressing cells, LC3 puncta were formed in the cytoplasm and most were colocalized with WT E2F3d, whereas LC3 puncta were barely observed in the LIR mutant E2F3d-expressing cells (Supplementary Fig. 3c). Given that expression of the LIR mutant E2F3d induced mitochondrial fragmentation to a similar extent as WT E2F3d (Fig. 3a), these results suggest that E2F3d induces mitochondrial fragmentation, independent of its interaction with LC3, like other mitophagy receptors^17,19^.

The expression levels of mitochondrial proteins, such as Tim23 and Tom20, were decreased by WT E2F3d, and this decrease was prevented by treatment with bafilomycin A1 (BFA), a specific inhibitor of lysosomal acidification and protein degradation (Fig. 3c). In addition, WT E2F3d enhanced the conversion of LC3-I to the lower migrating form LC3-II (Fig. 3c), which is associated with autophagosome formation^8^, and BFA treatment prominently increased LC3-II protein levels in WT E2F3d-expressing cells (Fig. 3d). Consistent with the decrease in the levels of mitochondrial proteins, mitochondrial DNA content also declined in WT E2F3d-expressing cells (Fig. 3e). When autophagic flux is promoted, an increased amount of LC3-II in the basal state is further increased by BFA treatment^22^. Although there is a possibility that E2F3d inhibits lysosomal degradation, these results suggest that E2F3d can accelerate mitochondrial turnover by promoting autophagic flux. Electron microscopy data showed that the WT E2F3d-expressing cells contained mitochondrion-like structures sequestered in the autophagic vesicles (Fig. 3f). We next examined whether the expression of E2F3d induced an interaction between mitochondria and lysosomes. In WT E2F3d-expressing cells, lysosome-associated membrane protein 1 (LAMP1)-positive vesicles were found to be accumulated between fragmented mitochondria, and some of these vesicles enclosed mitochondrial dots (Fig. 3g). To estimate the occurrence of mitophagy, cells were treated with Mtphagy Dye, which accumulates in mitochondria and emits high fluorescence when mitochondria fuse to lysosomes. The fluorescence intensity of Mtphagy Dye was observably increased by WT E2F3d, whereas it was only slightly enhanced by LIR mutant E2F3d (Fig. 3h). Collectively, these data demonstrate that WT E2F3d triggers the engulfment of fragmented mitochondria by autolysosomes.

During autophagosome formation, the ATG12–ATG5–ATG16L1 complex is recruited to the isolation membrane and facilitates the conjugation of LC3 proteins to phosphatidylethanolamine. This promotes their association with the isolation membrane and the subsequent maturation of the phagophore^23^. We, therefore, examined whether the ATG12–ATG5–ATG16L1 complex participates in E2F3d-induced mitophagy using ATG5 knockdown cells. The decrease in the levels of mitochondrial proteins and the conversion of LC3-I to LC3-II induced by WT E2F3d were both suppressed in these cells (Supplementary Fig. 4a). The expression of WT E2F3d was lower than that of the LIR mutant E2F3d in control cells, whereas the expression of both proteins was almost comparable in ATG5 knockdown cells (Supplementary Fig. 4b). These results imply that the expression of WT E2F3d is attenuated by the mitophagy itself induces. In contrast, and similar to control cells, mitochondrial fragmentation was induced by WT E2F3d in ATG5 knockdown cells (Supplementary Fig. 4c), suggesting that ATG5 is not essential for E2F3d-induced mitochondrial fragmentation.

### E2F3d is involved in hypoxia-induced mitophagy

We were unable to specifically knock down the expression of E2F3d using shRNA, and as such we knocked out *E2F3a*, *E2F3c*, and *E2F3d* simultaneously using CRISPR/Cas9-mediated genome editing. To this end, exon 1 of human *E2F3a* (exon 1a) was partially deleted from the genomic DNA. This deletion was expected to form an in-frame UAG stop codon adjacent to the translation initiation codon (Supplementary Fig. 5a). Partial deletion of exon 1a was first estimated by PCR-based genotyping and verified by DNA sequencing (Supplementary Fig. 5b, c). In E2F3a/c/d knockout (KO) cells, the protein expression of E2F3a, E2F3c, and E2F3d, but not E2F3b, was completely silenced (Supplementary Fig. 5d, e).

**Fig. 4:**
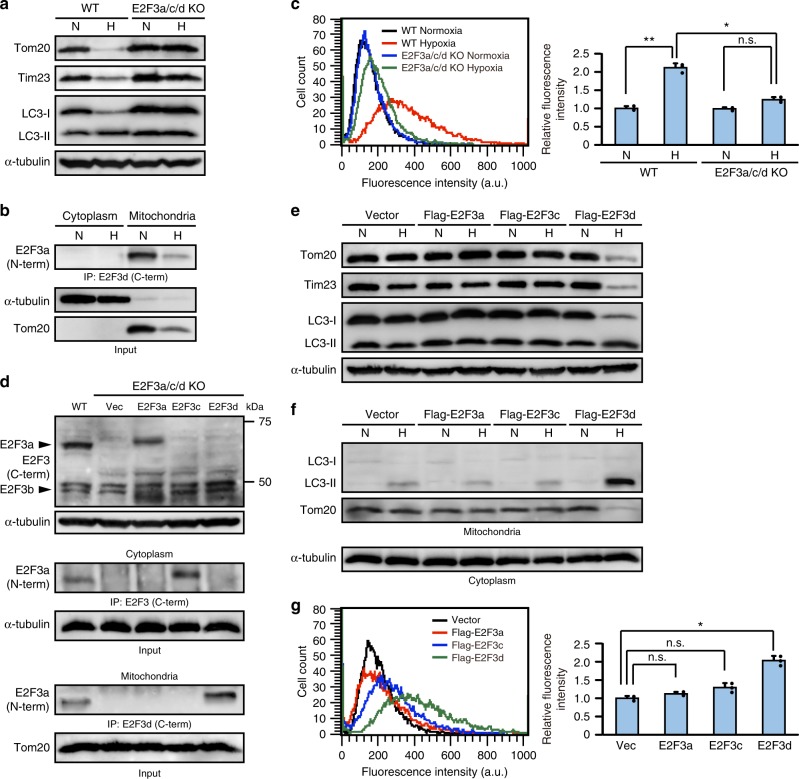
E2F3d is important for hypoxia-induced mitophagy. **a**–**c** Parental WT and E2F3a/c/d KO HeLa cells were cultured under normoxic (N; 21% O_2_) or hypoxic (H; 1% O_2_) conditions for 24 h. **a** Cell lysates were examined by immunoblotting. **b** Cytoplasmic and mitochondrial lysates from parental WT cells were immunoprecipitated with the antibody against the C-terminal domain of human E2F3d protein and probed with the antibody against the N-terminal domain of human E2F3a protein. **c** The uptake of mitochondria by lysosomes was evaluated by mitophagy assay. Data are presented as the mean of three independent experiments (≥10,000 cells). Error bars represent SD. *P < 0.05 and **P < 0.01. n.s. not significant, a.u. arbitrary units. **d**–**g** E2F3a/c/d KO HeLa cells were infected with control retrovirus (Vec) or retroviruses expressing Flag-E2F3a, Flag-E2F3c, or Flag-E2F3d. **d** Cell lysates were examined by immunoblotting (top). Cytoplasmic (middle) and mitochondrial (bottom) lysates were immunoprecipitated with the antibodies against the C-terminal domains of human E2F3a or E2F3d proteins, and probed with the antibody against the N-terminal domain of human E2F3a protein. **e**, **f** Cells were cultured under normoxic or hypoxic conditions for 24 h. Cell lysates (**e**) and cytoplasmic lysates and mitochondria (**f**) were examined by immunoblotting. **g** Cells were cultured under hypoxic conditions for 24 h and the uptake of mitochondria by lysosomes was analyzed as described in **c**. *P < 0.05. n.s. not significant. Full-size scans of immunoblots are shown in Supplementary Information. Please see Supplementary Fig. 5

**Fig. 5:**
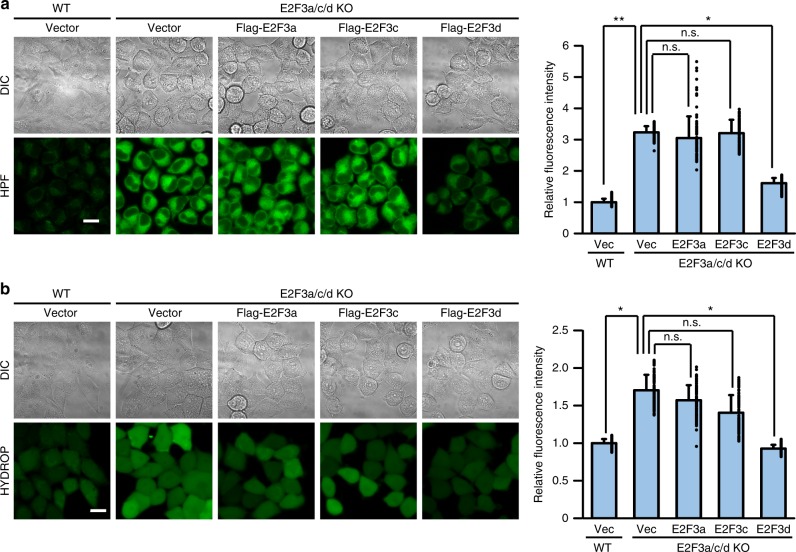
E2F3d affects intracellular ROS levels. **a**, **b** E2F3a/c/d KO HeLa cells were infected with control retrovirus (Vec) or retroviruses expressing Flag-E2F3a, Flag-E2F3c, or Flag-E2F3d. Intracellular ROS levels were analyzed using the fluorescent probes hydroxyl radical and peroxynitrite sensor HPF (**a**) and hydrogen peroxide sensor HYDROP (**b**). Differential interference contrast (DIC) and fluorescent images of HPF or HYDROP are shown. Scale bar, 20 μm. The mean fluorescence intensity was quantified (*n* = 200). Relative fluorescence intensities to parental WT cells infected with control retrovirus (Vec) are shown. Error bars represent SD. *P < 0.05 and **P < 0.01. n.s. not significant

Solid tumors often grow in hypoxic conditions because of insufficient functional vasculature, triggering mitophagy to selectively degrade damaged mitochondria and maintain mitochondrial quality^7^. When cells were cultured under hypoxic conditions, the levels of mitochondrial proteins, including E2F3d, decreased and the conversion of LC3-I to LC3-II increased in WT cells, whereas this decrease in mitochondrial protein levels and increase in LC3 conversion was compromised in E2F3a/c/d KO cells (Fig. 4a, b). Furthermore, hypoxia-induced mitochondria–lysosome fusion was diminished in E2F3a/c/d KO cells compared with that in WT cells (Fig. 4c). Next, we retrovirally transduced *E2F3a*, *E2F3c*, or *E2F3d* into E2F3a/c/d KO cells (Fig. 4d) and examined the occurrence of hypoxia-induced mitophagy to determine the importance of each E2F3 protein family member to this process. Hypoxia decreased the expression levels of mitochondrial proteins and promoted the conversion of LC3-I to LC3-II in E2F3d-rescued cells (Fig. 4e) and hypoxia-induced recruitment of LC3-II to mitochondria was also considerably enhanced (Fig. 4f), which might be attributed to the E2F3d–LC3 interaction. Consistently, mitochondria–lysosome fusion under hypoxic conditions was notably observed in E2F3d-rescued cells; however, it was weakly induced in E2F3a- or E2F3c-rescued cells (Fig. 4g). These results suggest that E2F3a and E2F3c play a minor role in hypoxia-induced mitophagy in cancer cells, and that E2F3d may be important for this process. Failure of mitochondrial quality control leads to the accumulation of damaged mitochondria, which is often associated with elevated levels of reactive oxygen species (ROS)^24^. ROS levels were observably increased in E2F3a/c/d KO cells, whereas this increase was reverted in E2F3d-rescued cells (Fig. 5a, b).

**Fig. 6:**
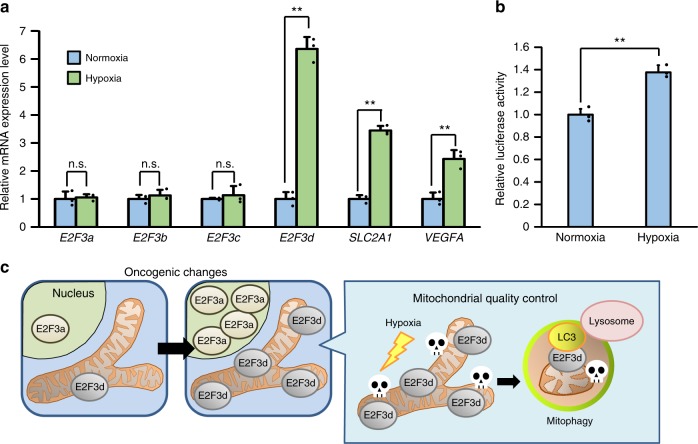
Hypoxia increases *E2F3d* transcripts. **a** HeLa cells were cultured under normoxic (21% O_2_) or hypoxic (1% O_2_) conditions for 24 h. cDNA samples were subjected to qRT-PCR using primer sets specific for each *E2F3* member and HIF-1 target genes. Values shown represent the means of three independent experiments. Error bars represent SD. **P < 0.01. n.s. not significant. **b** HeLa cells were co-transfected with an *E2F3a* promoter (−740 to +160) luciferase reporter plasmid and pCMV-RL. After 24 h of transfection, cells were cultured for a further 24 h under normoxic or hypoxic conditions. Values shown represent the means of three independent experiments. Error bars represent SD. **P < 0.01. **c** Model for E2F3d-mediated mitophagy for mitochondrial quality control in cancer cells

To further examine the effect of hypoxia on the regulation of E2F3d expression, we compared the transcription levels of *E2F3* family members under normoxic and hypoxic conditions as well as the hypoxia-inducible factor-1 (HIF-1) target genes, vascular endothelial growth factor A (*VEGFA*), and solute carrier family 2 member 1 (*SLC2A1*, also known as *GLUT1*) (Fig. 6a). The results revealed that hypoxia increased the transcripts of both *E2F3d* and HIF-1 target genes, but did not increase the transcripts of *E2F3a* and *E2F3c*, suggesting that splicing ratios of *E2F3a* transcripts were altered during hypoxia. We next examined whether the *E2F3a* promoter was activated during hypoxia and found that transcription from the *E2F3a* promoters was enhanced during hypoxia (Fig. 6b). These data indicate that hypoxia increases the expression of *E2F3d* through two possible mechanisms: by the alteration of the splicing ratios of *E2F3a* transcripts and/or the activation of the *E2F3a* promoter. Given that hypoxia participates in the regulation of alternative splicing events in cancer cells^25^, further studies are required to fully understand the regulation of *E2F3d* expression during hypoxia. Taken together, E2F3d is involved in hypoxia-induced mitophagy and mitochondrial quality control in cancer cells.

## Discussion

Genetic deletion of an essential autophagy gene alters the fate of Ras-driven tumors from adenocarcinomas to oncocytomas, the vast majority of which are benign and characterized by the accumulation of defective mitochondria^26^. These findings imply that mitophagy is essential for cancerous tumors, but not for benign tumors, and suggest that dysfunctional mitochondria may generate tumorigenic signals in benign tumors, such as increased production of ROS. In contrast, autophagy is elevated in Ras-activated cancer cells, and mitophagic clearance of dysfunctional mitochondria is required for intact mitochondrial metabolism, leading to growth and survival of these cancer cells^27^. Because overproduction of ROS by mitochondria can cause unrepaired nuclear DNA damage and subsequent apoptotic death in cancer cells^6,24^, mitochondrial quality control is important for cancer cell survival. Collectively, the anti- and pro-tumorigenic roles of mitophagy depend on the tumor stage.

Because mitochondrial function is critical for malignant tumor progression, a healthy mitochondrial population and mitochondrial quality control must be maintained during this process. Indeed, mechanisms for mitochondrial quality control, which are mainly achieved by the interplay between mitochondrial biogenesis and mitophagy, are often upregulated in cancer cells^28^. Mitochondrial biogenesis is primarily regulated by transcriptional programs that coordinate the expression of mitochondrial genes. Overexpression of Myc, one of the most common alterations in human cancers, is involved in the regulation of mitochondrial biogenesis^1^. Myc binds to the promoters of mitochondrial genes and increases in Myc function increase mitochondrial mass^29^. The *E2F3a* promoter possesses Myc-binding elements as well as E2F-binding elements, and overexpression of Myc protein stimulates the activity of the *E2F3a* promoter^30^. These findings suggest that overexpression of Myc may induce the expression of E2F3d, contributing to not only mitochondrial biogenesis but also E2F3d-induced mitophagy.

The regulation mechanisms of hypoxia-induced mitophagy mediated by E2F3d are currently unclear. It has been reported that FUNDC1 is involved in hypoxia-induced mitophagy, and tyrosine dephosphorylation in its LIR motif is intimately linked to its activity during this process^19^. Given that the amino acid sequence of the LIR motif in E2F3d is similar to that in FUNDC1 (Fig. 3b), E2F3d-mediated mitophagy under hypoxic conditions may be regulated in the same manner as FUNDC1.

The function of E2F3c is still unknown. It has been reported that E2F3a physically interacts with transcription factor E3 (TFE3) through a region located adjacent to the C-terminal transcriptional activation domain^31^ and most of this region is conserved in E2F3c. TFE3 predominantly localizes to the cytoplasm and its nuclear translocation is triggered under autophagy-inducing conditions such as nutrient starvation. Nuclear TFE3 enhances the expression of cellular genes that encode proteins involved in autophagy and lysosomal biogenesis^32^. Thus, E2F3c might be involved in TFE3-dependent transcriptional regulation of these genes through its possible interaction with TFE3 and conserved C-terminal transcriptional activation domain.

Mitochondrial integrity is often threatened during tumor progression, requiring the upregulation of mitochondrial quality control mechanisms. Given the strong association between E2F3a expression and tumor malignancy^12,15^, it is conceivable that the expression of E2F3d is increased in high-grade cancers, which may confer high susceptibility to hypoxia and create favorable conditions for mitophagy (Fig. 6c). In the present study, we unveiled that E2F3d localizes to the OMM and physically interacts with LC3, and that overexpression of E2F3d induces mitochondrial fragmentation and mitophagy. These results suggest that E2F3d is a previously unrecognized mammalian mitophagy receptor and a potential target for cancer therapy.

## Methods

### Cell lines and cell culture

HFFs, HeLa cells, and 293T cells were obtained from American Type Culture Collection (ATCC SCRC-1041, ATCC CCL-2, and ATCC CRL-3216). They were cultured in high glucose Dulbecco’s modified Eagle’s medium (DMEM; Wako) supplemented with 10% fetal bovine serum (FBS; Corning), 100 units ml^*−*1^ penicillin (Wako), and 100 μg ml^−1^ streptomycin (Wako) at 37 °C under 5% CO_2_. HFFs were brought to quiescence by serum starvation (0.1% FBS) for 72 h. Hypoxic conditions were achieved using a hypoxic chamber (Billups-Rothenberg) flushed with a pre-analyzed gas mixture of 1% O_2_, 5% CO_2_, and 94% N_2_ (Iwatani fine gas).

### Antibodies and reagents

Antibodies used in this study were obtained from the following suppliers: anti-cyclin A (1:500 for immunoblotting, sc-596), anti-E2F1 (1:500, sc-251), anti-GST (1:3000, sc-138), and anti-E2F3 (1:500, sc-878 and 1:500, sc-879) were from Santa-Cruz Biotechnology; anti-Flag (1:1000, #F7425 and #F1804) and anti-α-tubulin (1:2000, #T9026) were from Sigma-Aldrich; anti-E1A (1:2000, #554155), anti-Tim23 (1:1000, #611222), and anti-Tom20 (1:700, #612278) were BD Biosciences; anti-p14^ARF^ (1:500, K0084-3) and anti-Atg5 (1:500, PM050) were from MBL Life science; anti-Hsp70 (ADI-SPA-810) was from Enzo Life Sciences; anti-LC3B (1:2000, NB100-2220) was from Novus Biologicals; anti-Drp1 (1:1000, A303-410A) was from Bethyl Laboratories; and anti-LAMP1 (ab24170) was from Abcam. An anti-E2F3d polyclonal antibody against a peptide, PQPTSYSRLRTK, near the C-terminus was generated in rabbits and affinity-purified (1:100, Eurofins). BFA was purchased from Tocris Bioscience.

### Mitophagy assay

To detect mitophagy, a Mitophagy Detection Kit (Dojindo Molecular Technologies) was used as described previously^33^. In brief, cells were washed twice with DMEM and incubated with 100 nM Mtphagy Dye diluted in DMEM for 30 min at 37 °C. After incubation, cells were washed twice with DMEM and then mitophagy was induced. Subsequently, cells were trypsinized and fluorescence intensity of Mtphagy Dye was analyzed with a FACSCalibur equipped with CellQuest Pro software (Becton Dickinson) at 488 nm excitation and 650 nm longpass emission. The total fluorescence intensity of the cells was statistically tested using Tukey post-test.

### Sodium dodecyl sulfate polyacrylamide gel electrophoresis and immunoblotting

Cells were rinsed with ice-cold PBS and lysed in extraction buffer (25 mM HEPES, 150 mM CH_3_COOK, 2 mM EDTA, 0.1% NP-40, 10% glycerol, 1 mM dithiothreitol, Protease Inhibitor Cocktail [Sigma-Aldrich], Halt Phosphatase Inhibitor Cocktail [Thermo Scientific], pH 7.2). Equivalent protein quantities were subjected to sodium dodecyl sulfate polyacrylamide gel electrophoresis (SDS-PAGE). Alternatively, equal cell numbers were lysed directly in 1× Laemmli sample buffer (2% SDS, 60 mM Tris-HCl, 5% 2-mercaptoethanol, 10% glycerol, 0.01% bromophenol blue, pH 6.8), sonicated, and resolved by SDS-PAGE. Cytoplasmic lysates and mitochondria were obtained from an equal number of cells using a Mitochondria Isolation Kit (Thermo Scientific). Proteins in the gel were transferred to polyvinylidene difluoride membranes and immunoblotted with indicated first antibodies and appropriate horseradish peroxide-conjugated secondary antibodies (Promega). ImmunoStar LD (Wako) was used to detect protein bands according to the manufacturer’s instructions. Band intensity quantification of protein signals in immunoblotting was performed using ImageJ.

### Immunoprecipitation

Cytoplasmic lysates and mitochondria were obtained from an equal number of cells using the Mitochondria Isolation Kit (Thermo Scientific) and mitochondria were dissolved in UltraRIPA buffer (Biodynamics Laboratory). Cell lysates were immunoprecipitated with indicated antibodies and Protein G Sepharose 4 Fast Flow (GE Healthcare) or anti-Flag antibody-conjugated agarose beads (#A2220; Sigma-Aldrich) for 2 h under rotation at 4 °C. The precipitated complexes were washed three times with extraction buffer or UltraRIPA buffer and then analyzed by immunoblotting.

### Immunofluorescence microscopy

Cells were fixed with 4% paraformaldehyde in PBS for 30 min, permeabilized with 0.2% (v/v) Triton X-100/PBS for 5 min, and blocked with 1% bovine serum albumin/PBS for 30 min at room temperature. Subsequently, cells were incubated with anti-Flag (1:300, #F1804; Sigma-Aldrich), anti-Tom20 (1:300, #612278; BD Biosciences), anti-LC3B (1:200, NB100-2220; Novus Biologicals), anti-LAMP1 (1:300, ab24170; Abcam), or anti-Hsp70 (1:400, ADI-SPA-810; Enzo Life Sciences) antibodies for 2 h, and further incubated with Alexa Fluor 488/546-conjugated goat anti-mouse IgG or Alexa Fluor 488-conjugated goat anti-rabbit IgG antibodies (Molecular Probes) for 30 min at room temperature. Mitochondria were stained with MitoTracker Red CMXRos (Cell Signaling Technology) for 30 min at 37 °C before fixation. 4ʹ,6-diamidino-2-phenylindole (DAPI) was used for nuclear staining. Confocal images were taken using a Nikon A1Rsi microscope, equipped with oil-immersion objectives (×60 and ×100). Images were acquired using Nikon NIS-Elements imaging software.

### Electron microscopy

Cells were fixed with 2% paraformaldehyde and 2% glutaraldehyde in 0.1 M phosphate buffer (PB, pH 7.4) for 30 min at 4 °C and then fixed with 2% glutaraldehyde in 0.1 M PB overnight at 4 °C. After fixation, the samples were washed three times with 0.1 M PB, postfixed with 2% osmium tetroxide in 0.1 M PB for 1 h at 4 °C, and then dehydrated in a graded ethanol series (50%, 70%, 90%, and 100%). The samples were embedded in resins and the resins were polymerized for 48 h at 60 °C. The polymerized resins were ultra-thin sectioned at 70 nm using an ultramicrotome (Ultracut UCT, Leica) and the sections were mounted on copper grids. They were stained with 2% uranyl acetate for 15 min at room temperature, washed with distilled water, and then secondary-stained with Lead stain solution (Sigma-Aldrich) for 3 min at room temperature. The samples were visualized using a transmission electron microscope (JEM-1400Plus; JEOL Ltd.) at an acceleration voltage of 80 kV. Digital images were acquired with a CCD camera (EM-14830RUBY2; JEOL Ltd.).

### Identification of unrecognized *E2F3* cDNAs by RT-PCR

Total RNA was extracted using Isogen II (Nippon Gene). First-strand cDNAs were generated using a 1st Strand cDNA Synthesis Kit for RT-PCR [AMV] (Roche) with random hexamer primers. The cDNA samples were amplified by PCR with the following primers: the first PCR reaction, 5′-AAAGAGCAGGAGCGAGAG-3′ and 5′-AACACTGCATGACAGATGTT-3′; the second PCR reaction, 5′-CGGGATCCATGAGAAAGGGAATCCAG-3′ and 5′-CCGAATTCTCAACTACACATGAAGTC-3′. The RT-PCR products were resolved on 2% agarose gels and cloned into pCR XL-Topo vector (Topo XL PCR Cloning Kit; Invitrogen).

### qRT-PCR

First-strand cDNAs were synthesized with oligo (dT) primers from total RNA. qRT-PCR was carried out using KAPA SYBR FAST qPCR Kit Master Mix (KAPA Biosystems) and a Thermal Cycler Dice Real Time System Single (TaKaRa). ΔΔCt was calculated for each sample reaction using *GAPDH* and *ACTB* as internal controls (for Figs 1 and 6, respectively). The primer sets are listed in Table 1. After the last PCR cycle, each sample was subjected to thermal melting curve analysis to check for nonspecific product formation.

**Table 1:** List of qRT-PCR primers used in this study

Target gene	Forward primer	Reverse primer
*E2F3a*	CAGCCTCCTCTACACCAC	AGGTACTGATGACCGCTT
*E2F3b*	GAAATGCCCTTACAGCAGCA	ACCATCTGAGAGGTACTGATGAC
*E2F3c*	GCAGCCTCCTCTACACCA	CCAAATGTATTTGTAGGCTCGGA
*E2F3d*	CGGCAGCCTCCTCTACA	GGTTGAAGCCAAGTCGGA
*SLC2A1*	GCGGAATTCAATGCTGATGAT	CAGTTTCGAGAAGCCCATGAG
*VEGFA*	TCGGGCCTCCGAAACCAT	CCTGGTGAGAGATCTGGT
*ACTB*	TTTAATAGTCATTCCAAATATGAGA	ACATAATTTACACGAAAGCA
*GAPDH*	GGAGTCCACTGGCGTCTTCA	GAGGGGCCATCCACAGTCTT

### Quantification of mitochondrial DNA copy number

DNA was isolated from cell pellets using a GenElute mammalian genomic DNA miniprep kit (Sigma-Aldrich). A Human Mitochondrial DNA (mtDNA) Monitoring Primer Set (Takara) and KAPA SYBR FAST qPCR Kit Master Mix (KAPA Biosystems) were used for quantitative PCR in conjunction with the Thermal Cycler Dice Real Time System Single (TaKaRa). Mitochondrial DNA copy number was then analyzed according to the manufacturer’s instructions.

### Proteinase K protection assay

Mitochondria were isolated using the Mitochondria Isolation Kit (Thermo Scientific) according to the manufacturer’s instructions. Isolated mitochondria were then suspended in MB buffer (220 mM mannitol, 70 mM sucrose, 10 mM HEPES, pH 7.5) and incubated on ice with proteinase K (50 μg ml^−1^) in the absence or presence of 1% Triton X-100 for 30 min. Digestion was terminated with 2 mM phenylmethylsulfonyl fluoride (final concentration). Mitochondrial proteins were separated by SDS-PAGE, followed by immunoblotting.

### Plasmid construction

To prepare the expression vectors used in the BiFC assay, cDNAs encoding the N-terminal (amino acids 1–173) and C-terminal (amino acids 155–239) fragments of Venus were fused upstream of sequences encoding Flag-tagged E2F3d deletion mutants and Hsp70 via linker sequences for SGLRS, respectively. Retroviral vectors that encode shRNAs against human *Drp1* were constructed by cloning suitable oligonucleotide sequences (shDrp1 #1, 5′-CGGTTCATCAGTAATCCTAAT-3′; shDrp1 #2, 5′-GCTACTTTACTCCAACTTATT-3′) into the pSUPER retro puro vector (Oligoengine). pBABE-puro Drp1 K38A was generated by subcloning *Drp1 K38A* cDNA from pcDNA3-Myc-Drp1 K38A into the pBABE-puro retroviral vector. Detailed information on all plasmids is available upon request.

### Retroviral infection

pSUPER retro puro shDrp1 #1, pSUPER retro puro shDrp1 #2, pBABE-puro Drp1 K38A, pBABE-puro E1A, pBABE-puro Flag-E2F3a, pBABE-puro Flag-E2F3c, and pBABE-puro Flag-E2F3d were used in this study. Retroviral constructs were transfected into 293 T cells together with amphotropic packaging plasmid. Supernatants were collected 48 h after transfection and filtered through a 0.45-μm filter. The supernatants containing virus were mixed with 4 μg ml^−1^ polybrene (final concentration) and added to cells seeded onto six-well plates. The plates were centrifuged at 450 × *g* for 1 h at 30 °C and then incubated overnight at 37 °C. Infected cell populations were selected using puromycin (1.5 μg ml^−1^ for HFFs and 2.0 μg ml^−1^ for HeLa cells) for 3 days.

### Infection with recombinant adenoviruses

Control recombinant adenovirus and recombinant adenoviruses expressing Flag-tagged WT or LIR mutant E2F3d were generated using the ViraPower adenoviral expression system (Life Technologies) according to the manufacturer’s protocol. Cells were infected with the recombinant adenoviruses for 24 h at a multiplicity of infection of 100.

### CRISPR/Cas9

pSpCas9(BB)-2A-Puro (PX459) v2.0 was a gift from Dr. Feng Zhang (Addgene plasmid #62988). CRISPR/Cas9-mediated genome editing was performed as described previously with minor modifications^34^. Oligonucleotide inserts that included a guiding RNA sequence were designed as follows: for guiding RNA #1, 5′-CACCGCTGCAGCCTCCATGGACAAA-3′ and 5′-AAACTTTGTCCATGGAGGCTGCAGC-3′; for guiding RNA #2, 5′-CACCGGGTACTGCTCCAGAGCGGGC-3′ and 5′-AAACGCCCGCTCTGGAGCAGTACCC-3′. After annealing, these oligonucleotides were inserted into the *Bbs*I cloning site. The two plasmids were co-transfected and the transfected cells were selected using puromycin (2.0 μg ml^−1^) for 3 days. Single colonies of the transfected cells were isolated with cloning cylinders and expanded for experiments. Genomic DNAs were extracted with the GenElute mammalian genomic DNA miniprep kit (Sigma-Aldrich), and PCR-based genotyping analysis was performed with the following primers: 5′-AAAGAGCAGGAGCGAGAG-3′ and 5′-TGAGGATCTGGATGT-3′.

### siRNA transfection

Cells were transfected with siRNA targeting human *ATG5* (siGENOME Human ATG5 siRNA-SMARTpool, M-004374-04; Dharmacon) or control siRNA (siGENOME Non-Targeting siRNA #1, D-001210-01; Dharmacon) at a final concentration of 10 nM using Lipofectamine RNAi MAX (Invitrogen).

### Luciferase assay

We used an *E2F3a* promoter (−740 to +160) luciferase reporter plasmid. As an internal control, a CMV promoter-driven *Renilla* luciferase construct (pCMV-RL) was co-transfected. Luciferase and *Renilla* luciferase activities were measured using the Dual-Luciferase Reporter Assay System (Promega), and luciferase activity was normalized to *Renilla* luciferase activity.

### GST pull-down assay

To obtain purified E2F3d proteins, Flag-tagged WT and LIR mutant E2F3d constructs were transfected into 293 T cells for 24 h and cell lysates were extracted. The Flag-E2F3d proteins were captured on anti-Flag antibody-agarose beads (#A2220; Sigma-Aldrich), washed three times with extraction buffer, and eluted by competition with free 1× Flag peptide (Sigma-Aldrich) dissolved in 50 mM Tris (pH 7.4) and 50 mM NaCl. Large amounts of eluent were pooled and concentrated with Amicon Ultra centrifugal filter 3K membrane (Millipore) because of the poor solubility of E2F3d by extraction buffer. GST-tagged proteins were expressed in *Esherichia coli* strain BL21(DE3)pLysS (Promega) by adding 200 μM isopropyl β-d-thiogalactopyranoside overnight at 18 °C. GST fusion proteins were purified on Glutathione Sepharose 4B (GE Healthcare). For the GST pull-down assay, 10 μg of GST fusion proteins were incubated with 1 μg of Flag-E2F3d proteins in 500 μl GST buffer (50 mM Tris-HCl, 150 mM NaCl, 5 mM EDTA, 0.5% NP-40, 1 mM dithiothreitol, Protease Inhibitor Cocktail [Sigma-Aldrich], pH 7.5) for 2 h under rotation at 4 °C and washed three times with GST buffer. The precipitate was analyzed by SDS-PAGE, followed by immunoblotting.

### Detection of ROS formation

Cells were treated with 10 μM hydroxyphenyl fluorescein (HPF; Goryo Chemical) or 5 μM HYDROP (Goryo Chemical) and then incubated for 30 min at 37 °C in the dark. After washing with PBS, the fluorescence images were obtained using a confocal fluorescence microscope (LSM700; Zeiss) at an excitation wavelength of 488 nm. The mean fluorescence intensity of HPF or HYDROP was quantified using ImageJ.

### Statistical analysis

Results are presented as the mean of independent experiments. Error bars represent SD. Statistical significance was evaluated using paired Student’s *t*-tests. One-way or two-way analysis of variance (ANOVA) followed by Tukey post-test was used to analyze the differences between multiple experimental groups. A *P*-value of <0.05 was considered statistically significant, and the *P*-values are represented in the figures as *P < 0.05 and **P < 0.01.

## Supplementary information

Supplementary Information

## Data Availability

The authors declare that all the data used in this study are available within the article and its Supplementary Information files or from the corresponding author upon reasonable request. The sequences for *E2F3c* and *E2F3d* reported here have been deposited in the Genbank (Accession numbers MK185232 and MK185233).
