# Supplementary material for: Mitochondrial protein E2F3d, a distinctive E2F3 product, mediates hypoxia-induced mitophagy in cancer cells
Source: Commun Biol. 2019 Jan 3;2:3. doi: 10.1038/s42003-018-0246-9 (PMC6318215; doi:10.1038/s42003-018-0246-9)

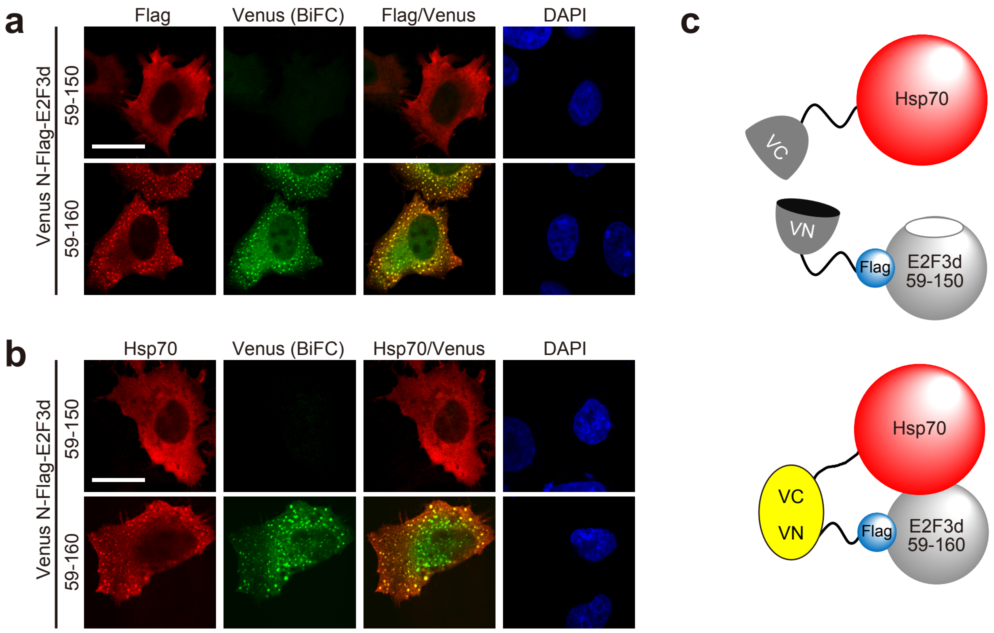

Supplementary Figure 1. E2F3d interacts with Hsp70. **a**, **b** HeLa cells were co-transfected with constructs for Flag-tagged E2F3d deletion mutants fused to the N-terminal fragment of Venus (VN) and Hsp70 fused to the C-terminal fragment of Venus (VC). Cells were then immunostained with anti-Flag antibody (**a**) or anti-Hsp70 antibody (**b**). Scale bar, 20 μm. **c** Schematic representation of the BiFC-negative and BiFC-positive combination of E2F3d deletion mutants and Hsp70.

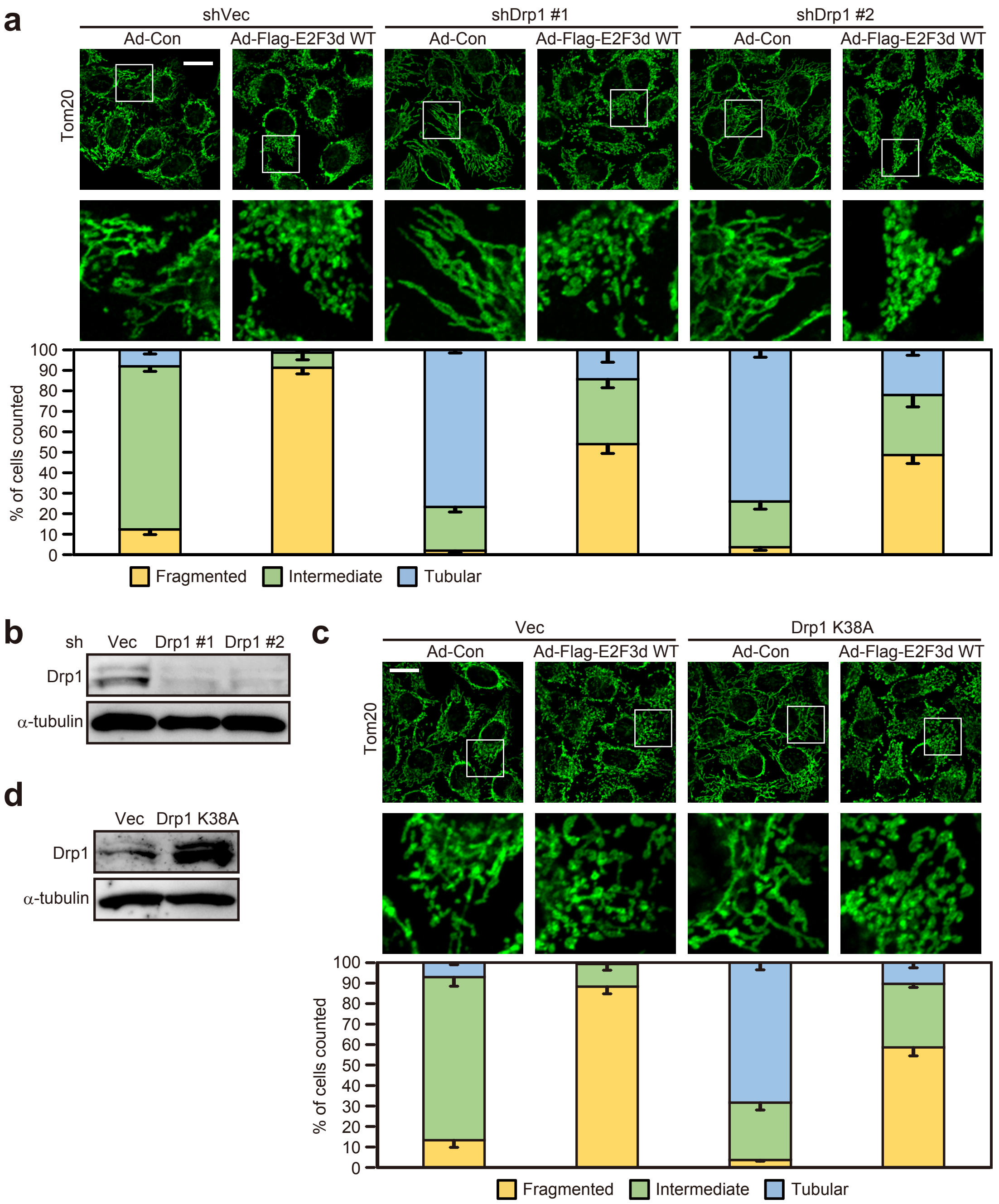

Supplementary Figure 2. Drp1 does not play a crucial role in E2F3d-induced mitochondrial fragmentation. **a**, **b** HeLa cells were infected with control shRNA retrovirus (shVec) or retroviruses expressing shRNA against human *Drp1* (shDrp1 #1 or shDrp1 #2). **a** Cells were infected with control adenovirus (Ad-Con) or adenovirus expressing Flag-tagged WT E2F3d (Ad-Flag-E2F3d WT) and then immunostained with anti-Tom20 antibody. The bottom images show magnifications of the boxed areas in the top images. Scale bar, 20 μm. The graph shows the percentage of cells exhibiting fragmented, intermediate, or tubular mitochondrial morphologies. Data are presented as the mean of three independent experiments (≥100 cells). Error bars represent SD. **b** Depletion of Drp1 protein was verified by immunoblotting. **c**, **d** HeLa cells were infected with control retrovirus (Vec) or retrovirus expressing a dominant-negative Drp1 mutant (Drp1 K38A). **c** Cells were analyzed as described in **a**. **d** The expression of Drp1 protein was examined by immunoblotting.

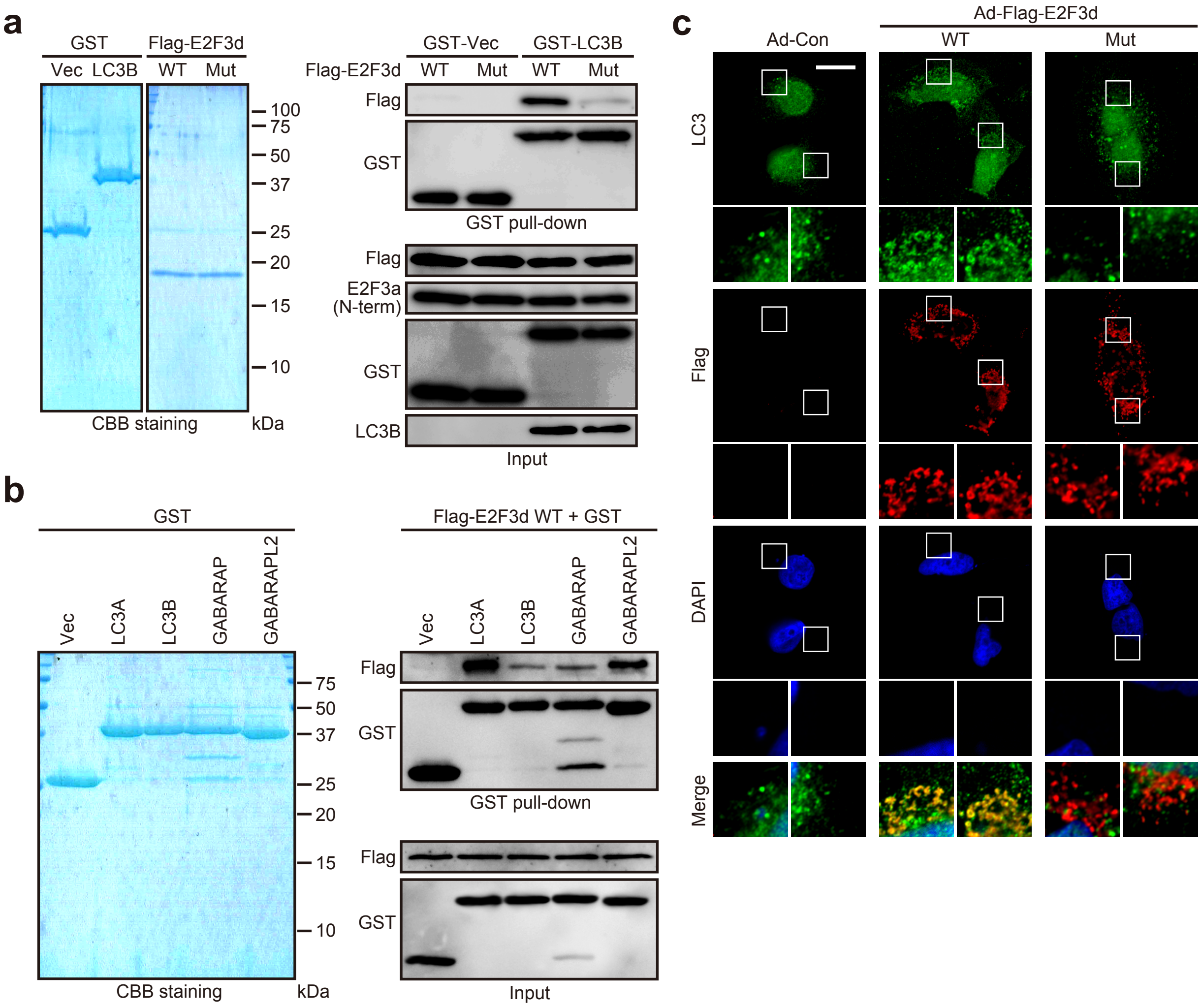

Supplementary Figure 3. E2F3d interacts with LC3. **a** The purity of GST and GST-LC3B proteins and Flag-tagged WT and LIR mutant (Mut) E2F3d proteins were evaluated by SDS-PAGE, followed by Coomassie Brilliant Blue (CBB) staining (left). Purified Flag-tagged E2F3d proteins were incubated with immobilized GST or GST-LC3B beads and the co-precipitated proteins were analyzed by immunoblotting (right). **b** GST fusion proteins of human ATG8 homologs were analyzed as described in **a**. **c** HeLa cells were infected with control adenovirus (Ad-Con) or adenoviruses expressing Flag-tagged WT or LIR mutant E2F3d (Ad-Flag-E2F3d WT or Mut). Cells were immunostained with anti-Flag and anti-LC3 antibodies. The boxed areas are shown at higher magnification. Scale bar, 20 μm.

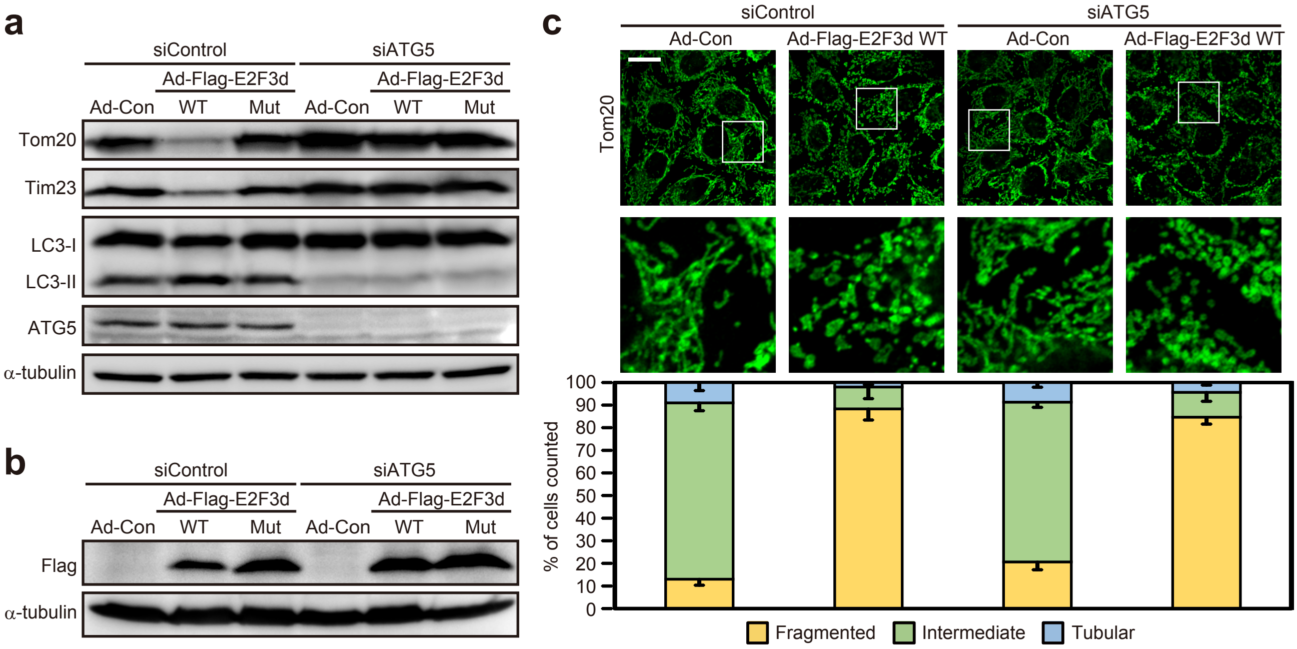

Supplementary Figure 4. E2F3d-induced mitophagy is dependent on ATG5. HeLa cells were transfected with control siRNA or siRNA against human *ATG5*. After 24 h, they were infected with control adenovirus (Ad-Con) or adenoviruses expressing Flag-tagged WT or LIR mutant E2F3d (Ad-Flag-E2F3d WT or Mut). **a** Cell lysates were examined by immunoblotting. **b** Cells were lysed directly in 1× Laemmli sample buffer and loaded onto SDS-polyacrylamide gels for immunoblotting. **c** Cells were immunostained with anti-Tom20 antibody. The bottom images show magnifications of the boxed areas in the top images. Scale bar, 20 μm. The graph shows the percentage of cells exhibiting fragmented, intermediate, or tubular mitochondrial morphologies. Data are presented as the mean of three independent experiments (≥100 cells). Error bars represent SD.

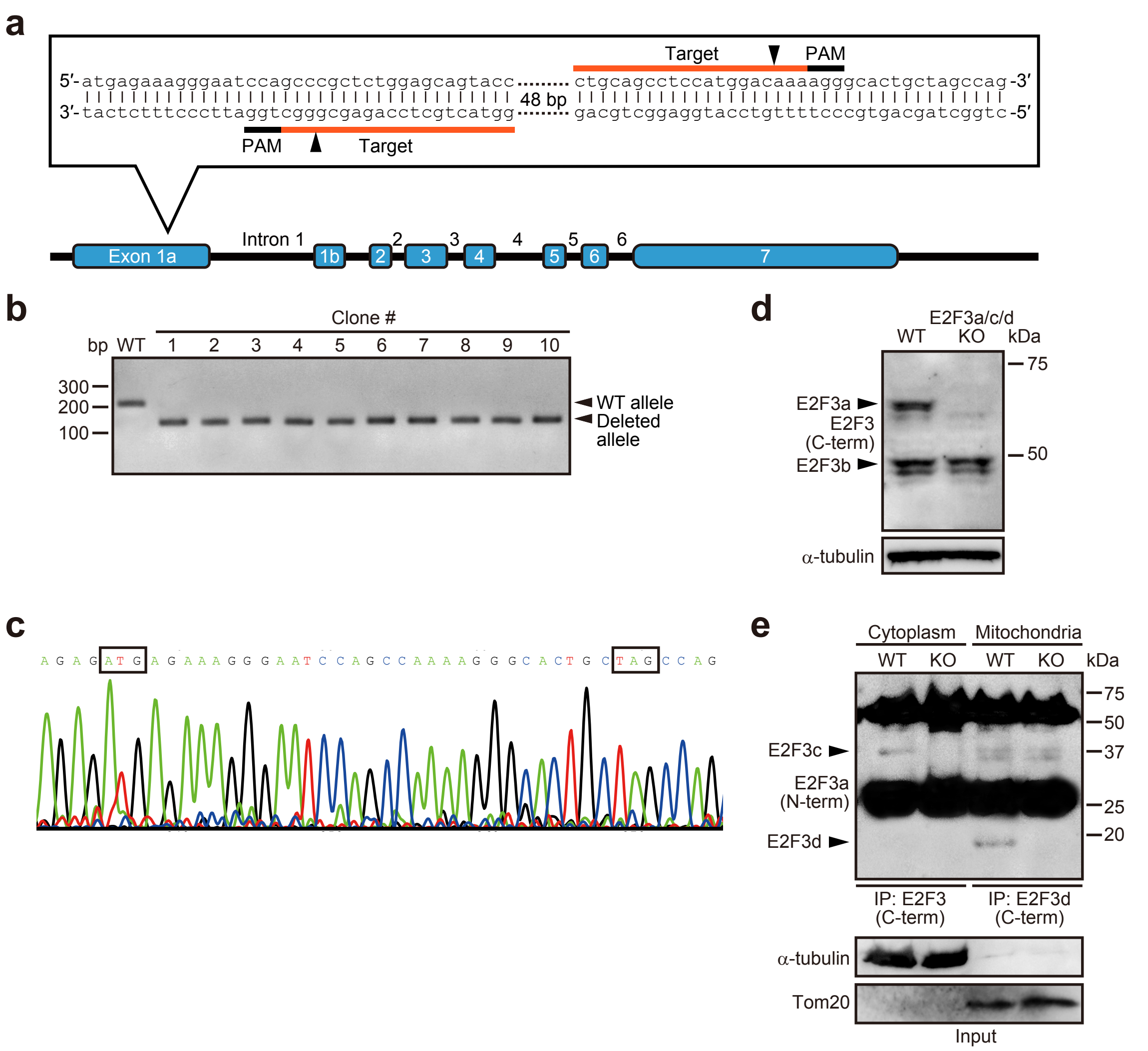

Supplementary Figure 5. The E2F3a/c/d KO cell line generated using the CRISPR/Cas9 system. **a** Schematic representing the design of the genome editing experiments for E2F3a/c/d KO. Arrowheads denote putative cleavage sites. **b** Genomic DNA was extracted from 10 single cell clones transfected with the PX459 plasmid expressing gRNAs against exon 1 of human *E2F3a* (exon 1a). PCR-based genotyping analysis was performed. Arrowheads indicate PCR products corresponding to the WT allele and exon 1a-deleted allele. WT shows genomic DNA from parental HeLa cells. **c** The PCR products in **b** were subjected to DNA sequencing to confirm the sequence of the potential deletion region. The translation initiation codon and in-frame stop codon are indicated in boxes. **d** Cell lysates from parental WT and E2F3a/c/d KO cells were examined by immunoblotting. **e** Cytoplasmic and mitochondrial lysates from parental WT and E2F3a/c/d KO cells were immunoprecipitated with the antibodies against the C-terminal domains of human E2F3a or E2F3d proteins, and probed with the antibody against the N-terminal domain of human E2F3a protein.

Full-size scans of immunoblots

Figure 1a

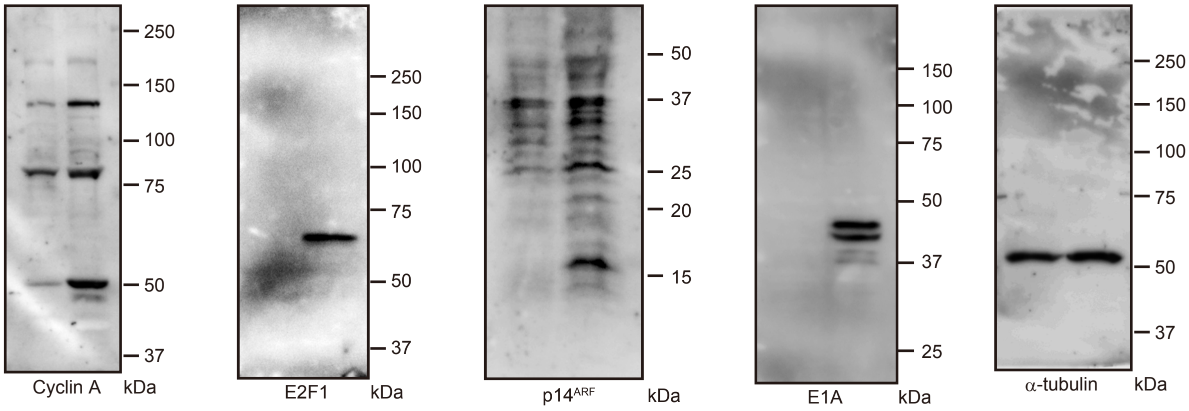

Figure 2d

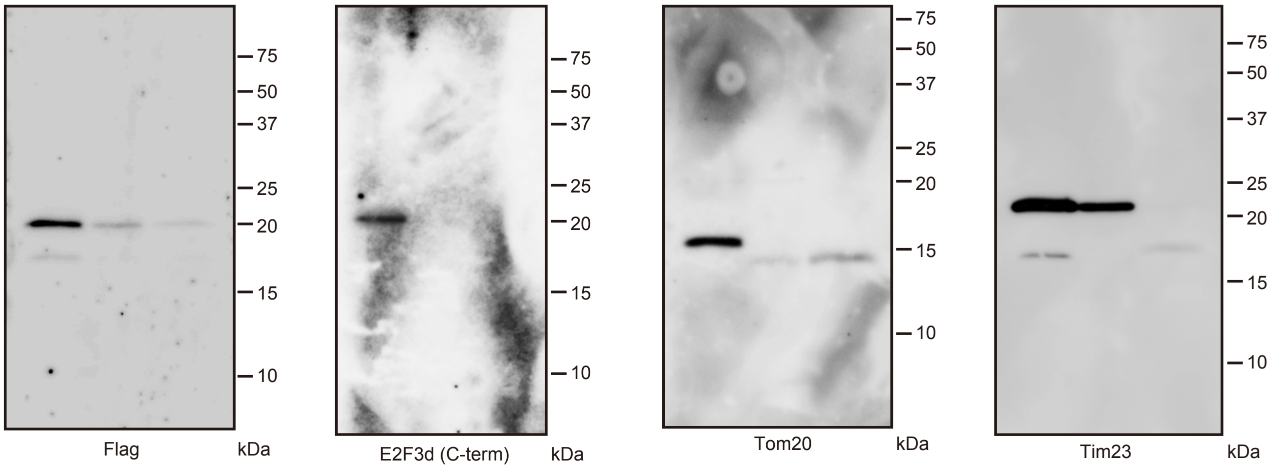

Figure 3c

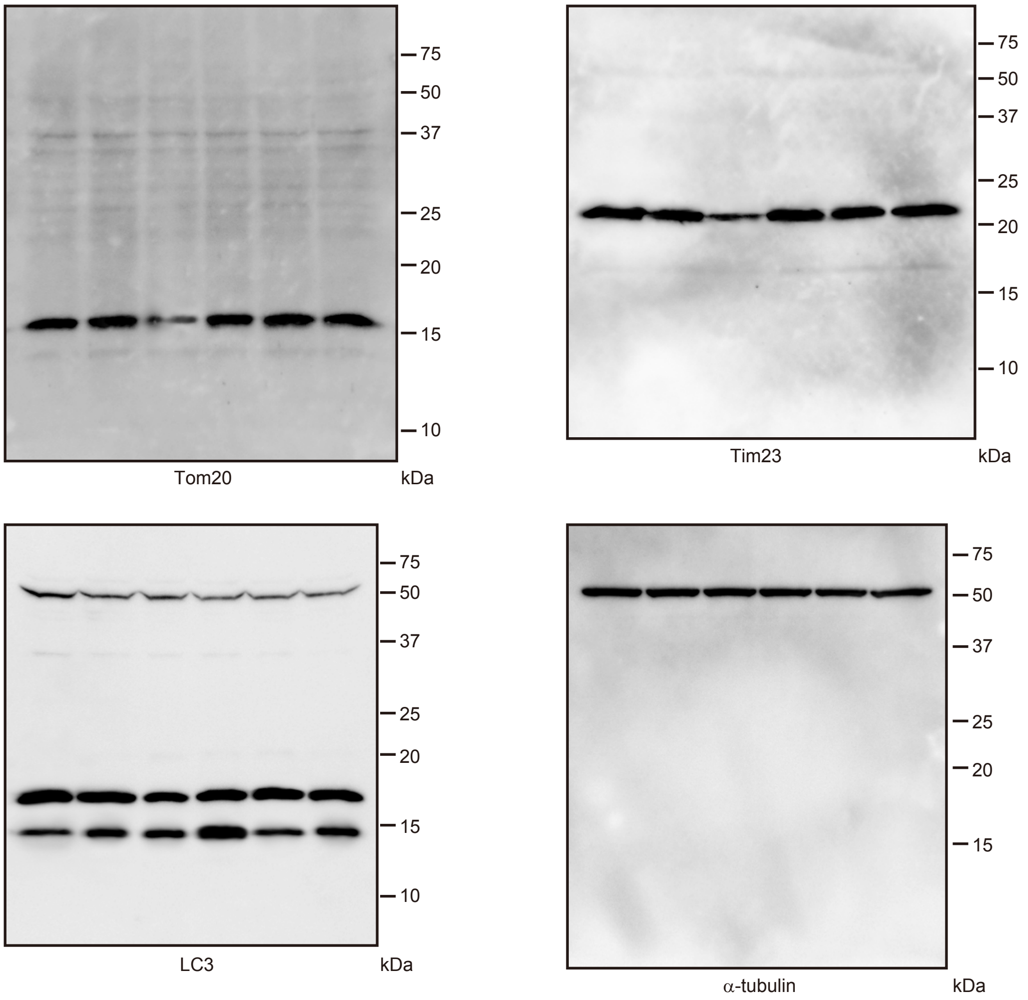

Figure 4a

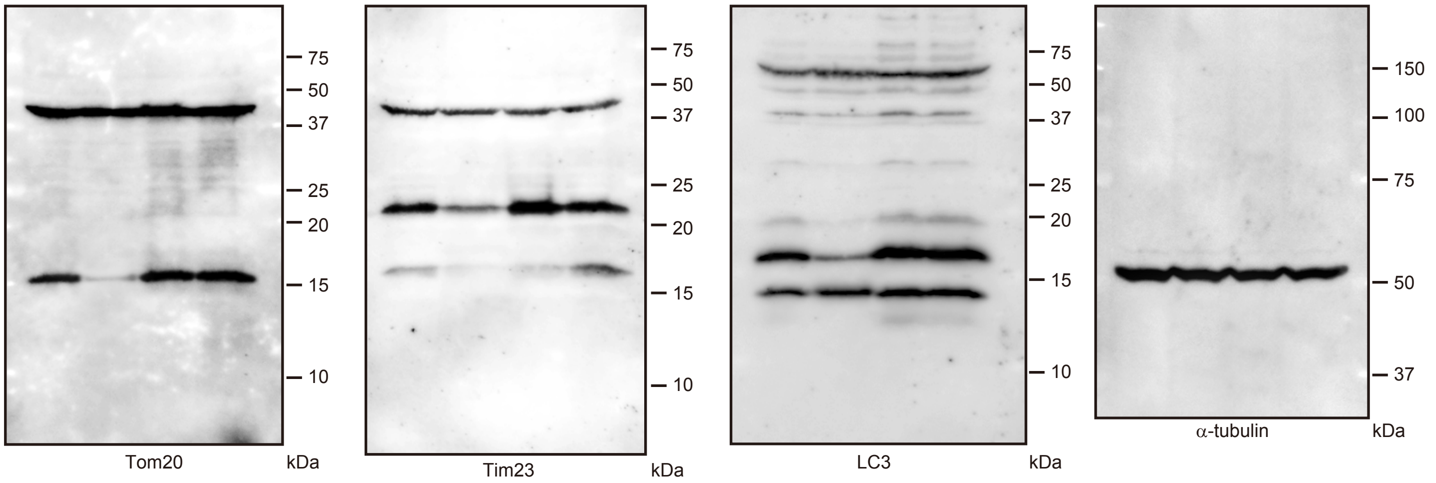

Figure 4b

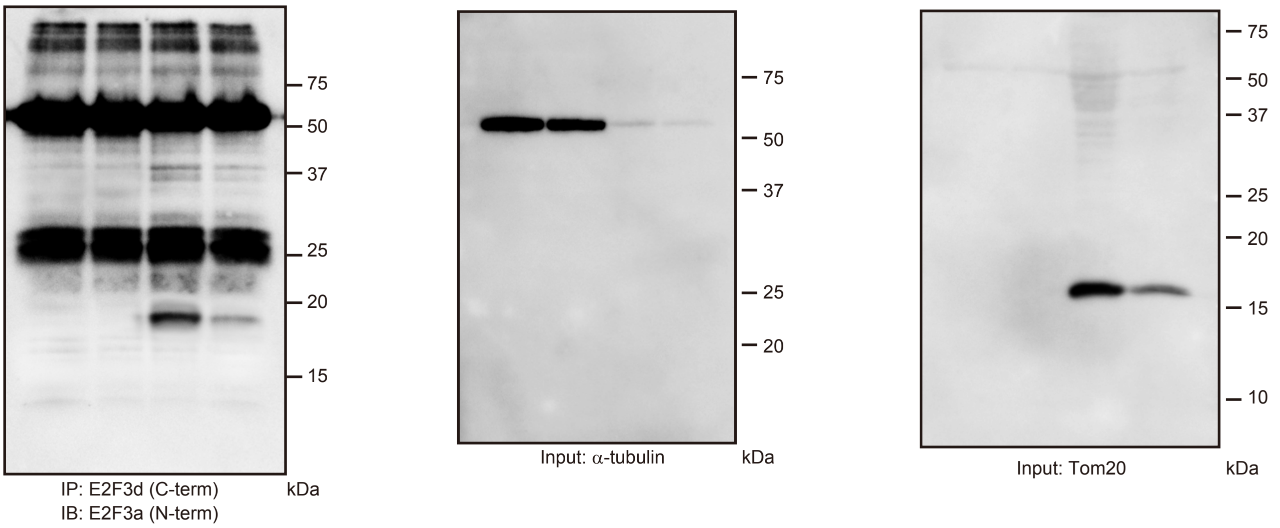

Figure 4d

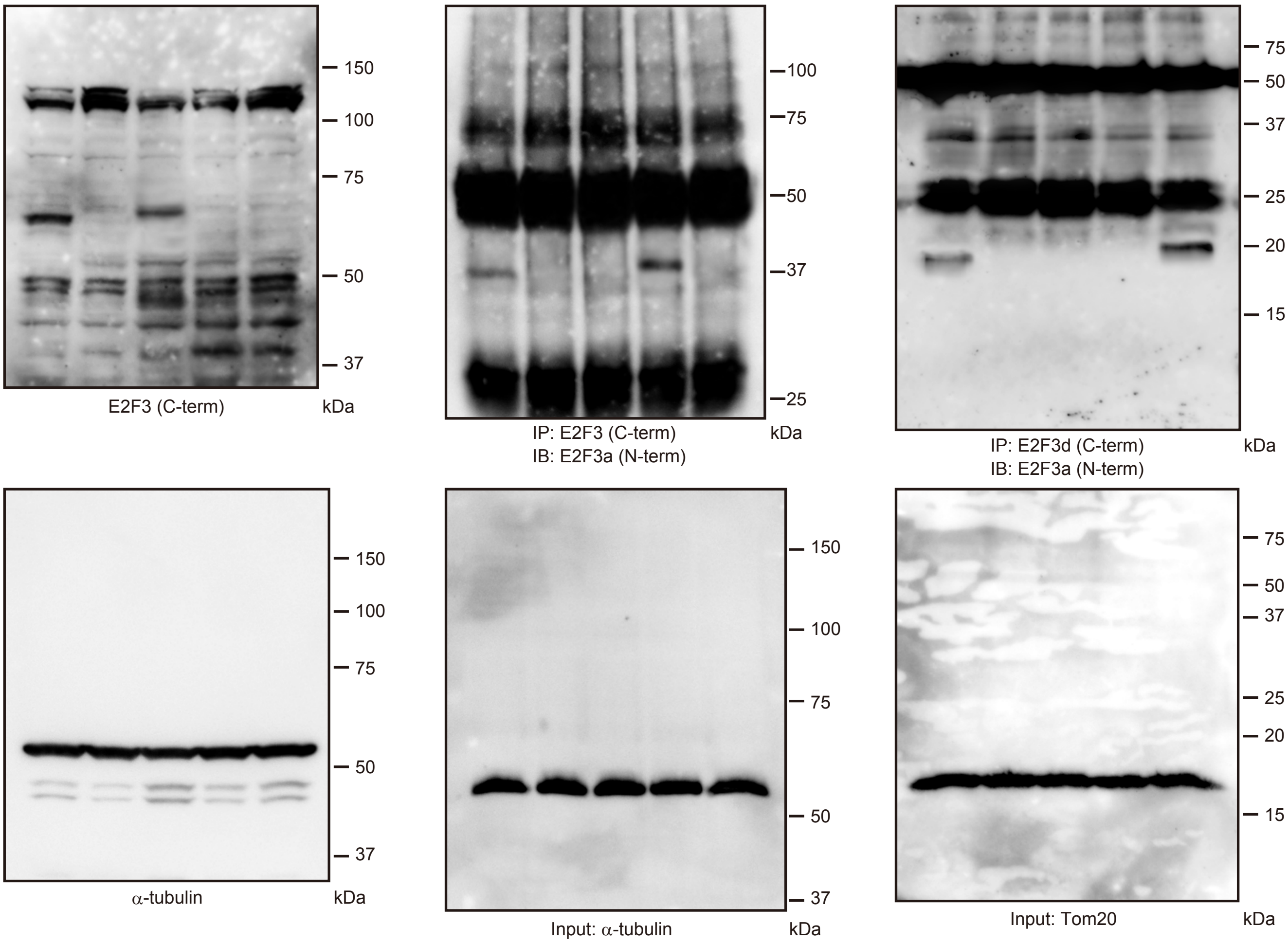

Figure 4e

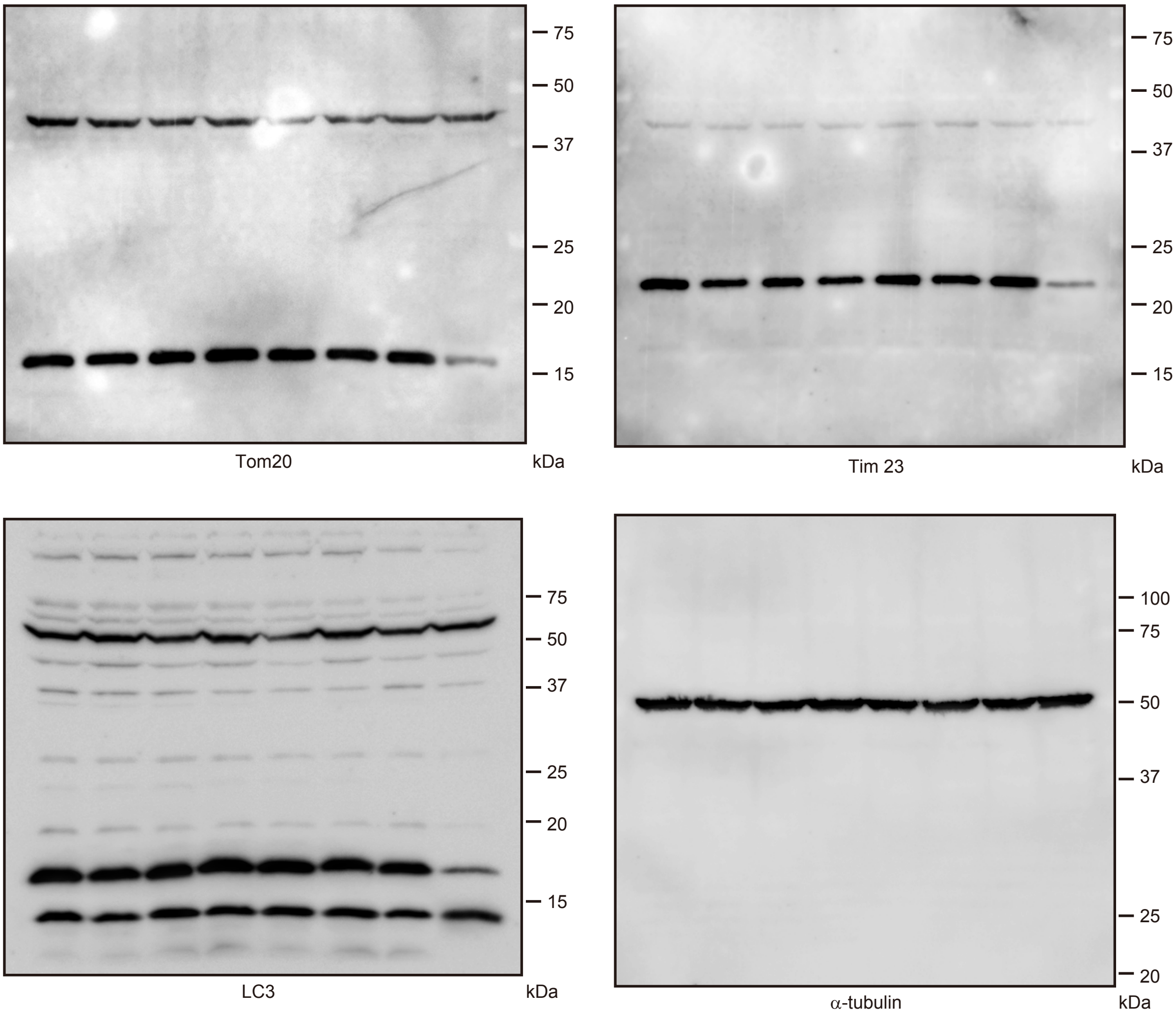

Figure 4f

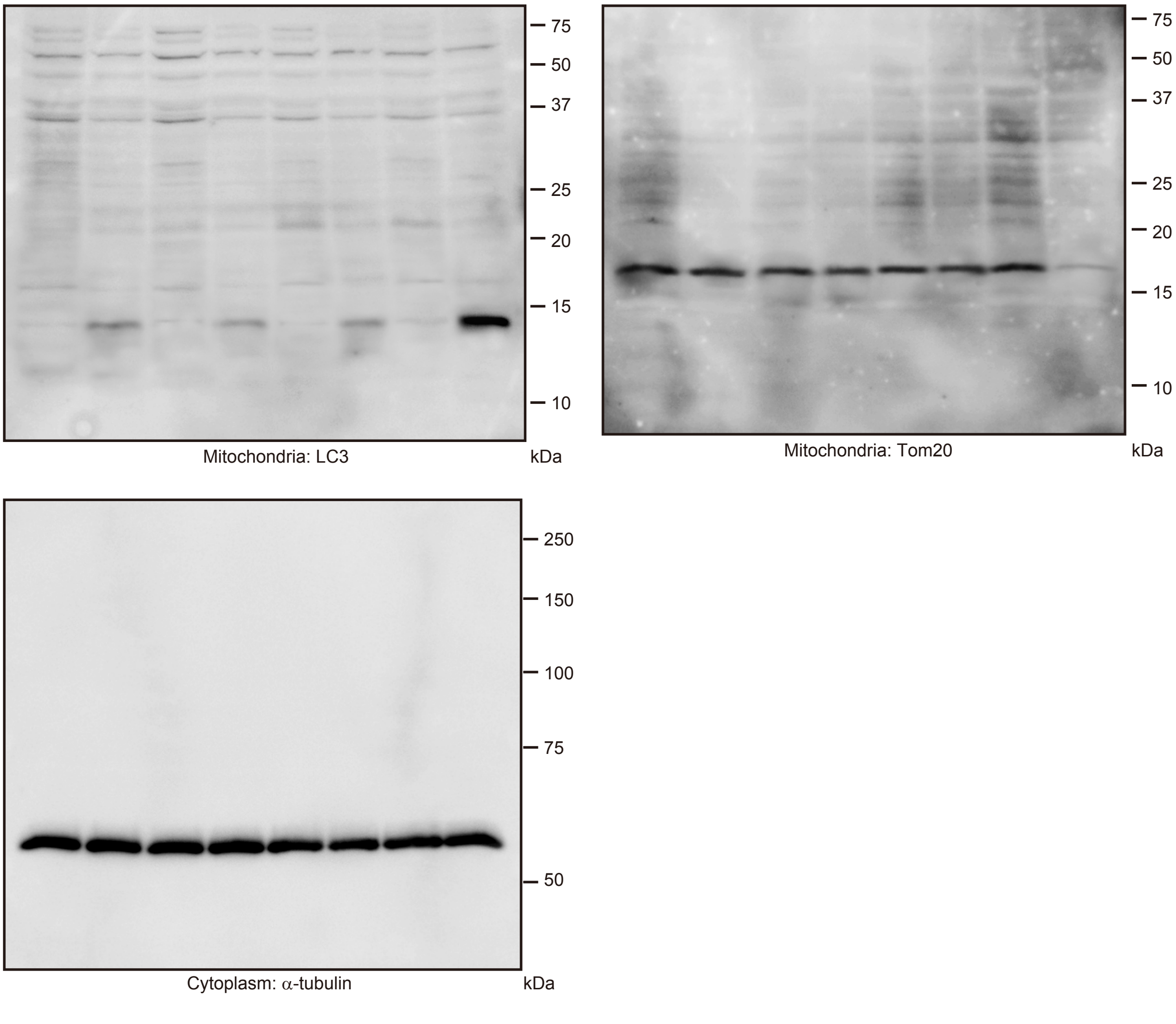

Supplementary Figure 2b

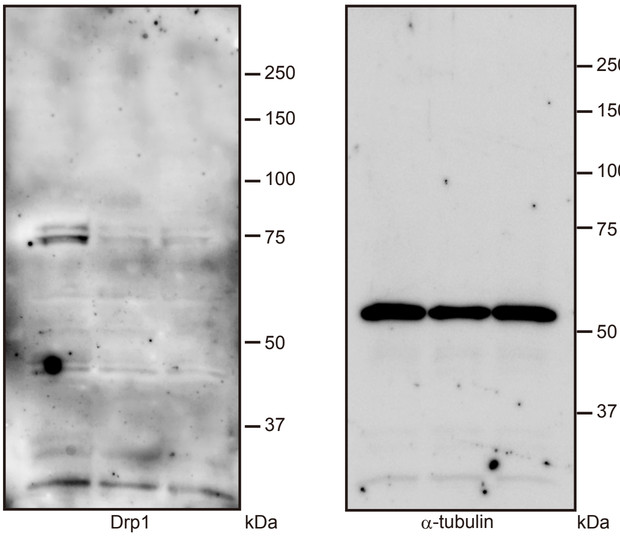

Supplementary Figure 2d

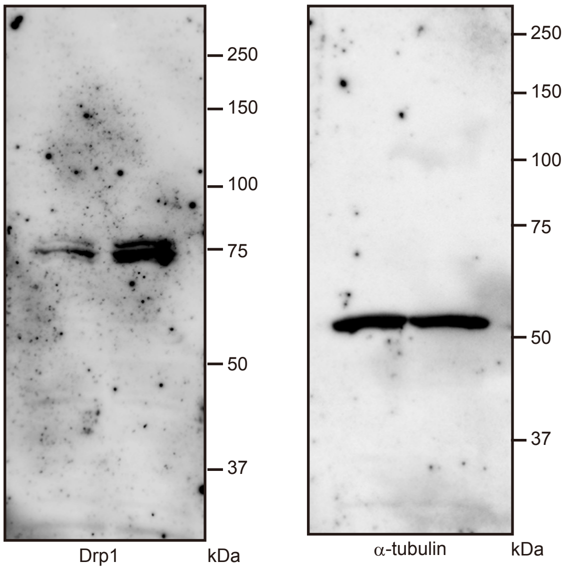

Supplementary Figure 3a

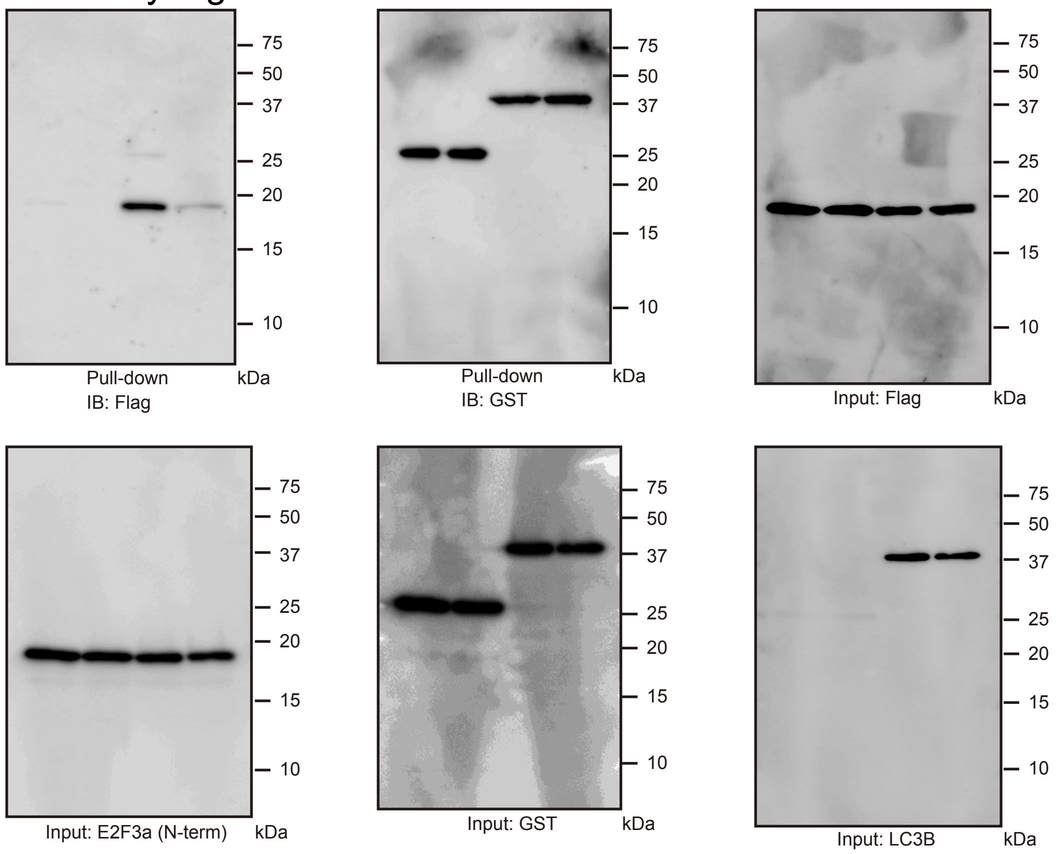

Supplementary Figure 3b

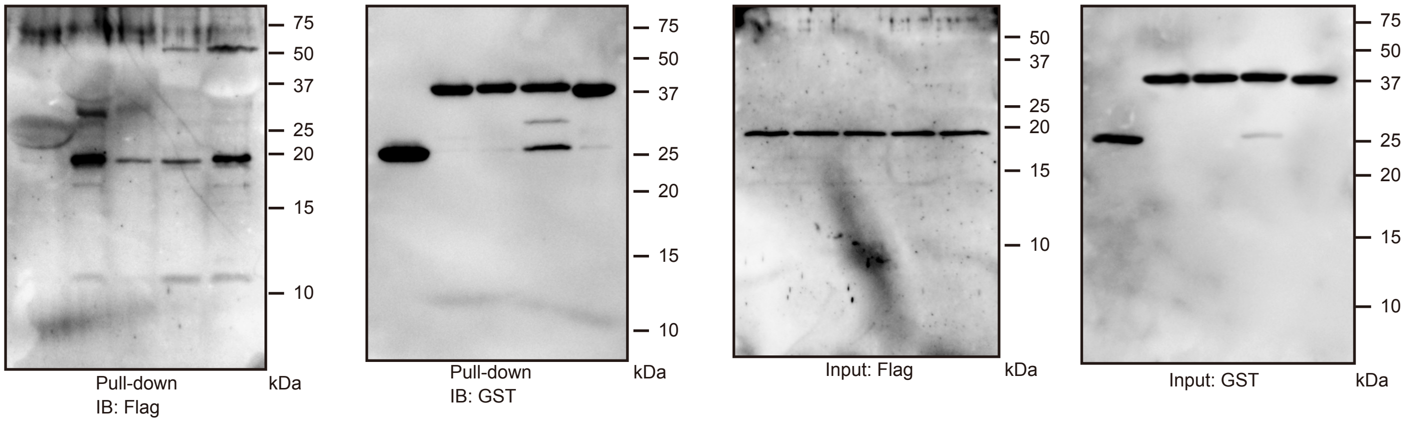

Supplementary Figure 4a

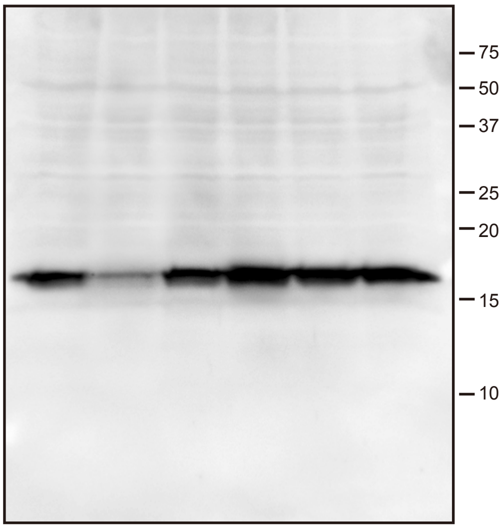

Tom20 kDa

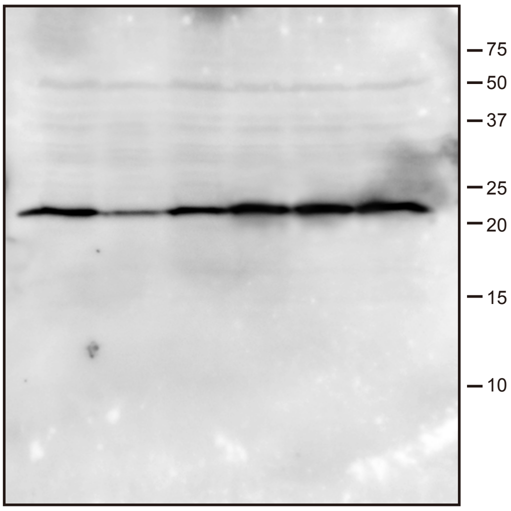

Tim23 kDa

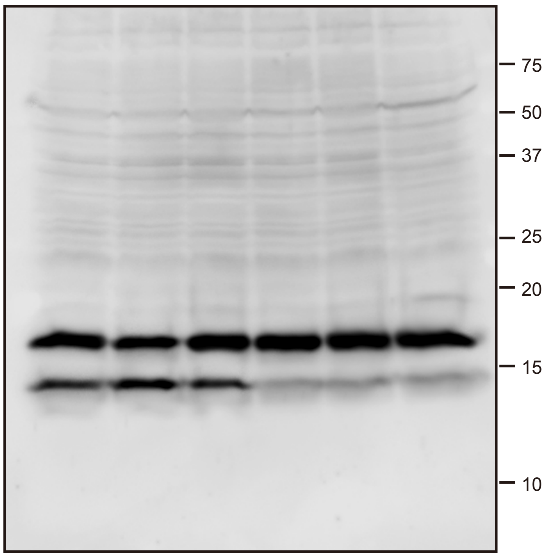

LC3 kDa

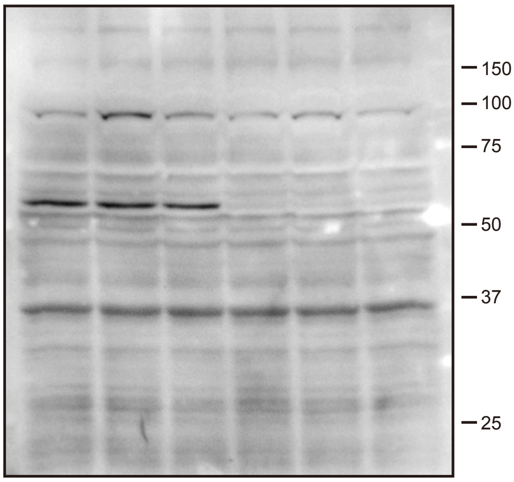

ATG5 kDa

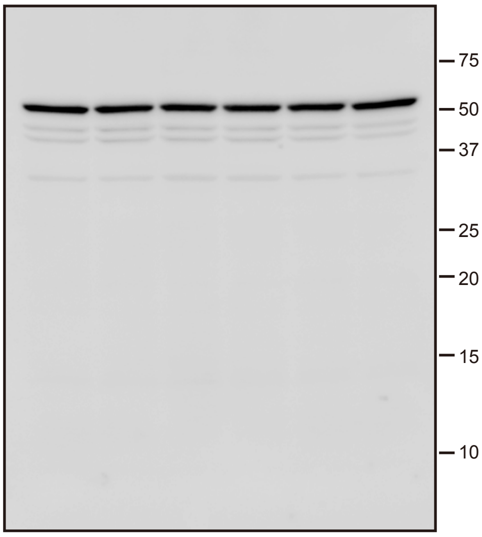

α-tubulin kDa

Supplementary Figure 4b

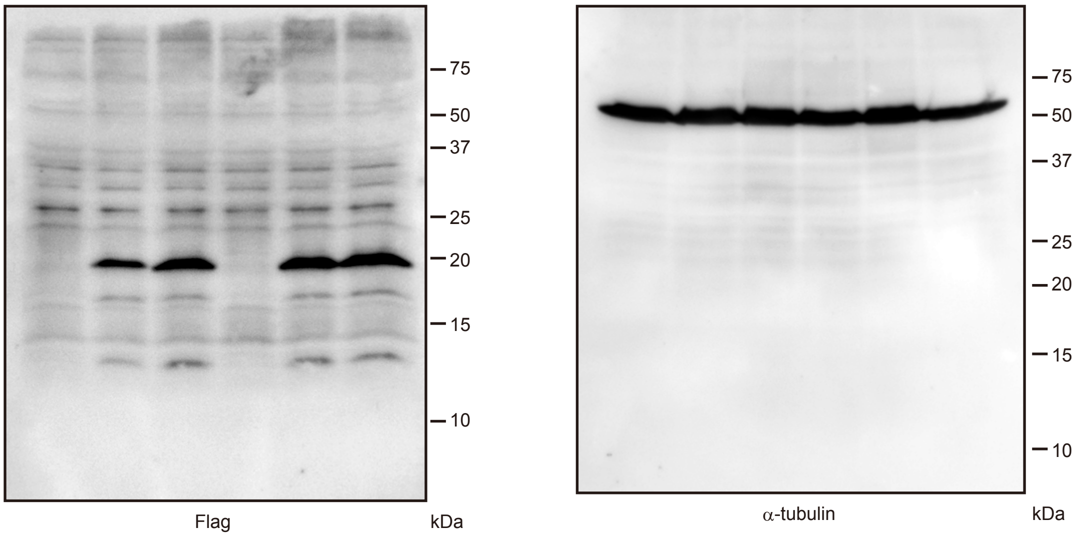

Supplementary Figure 5d

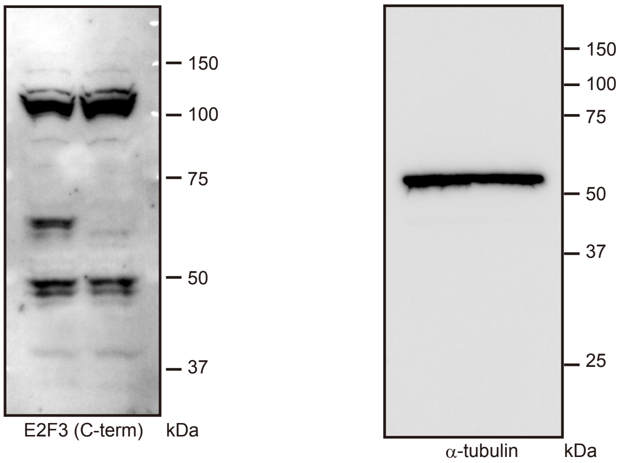

Supplementary Figure 5e

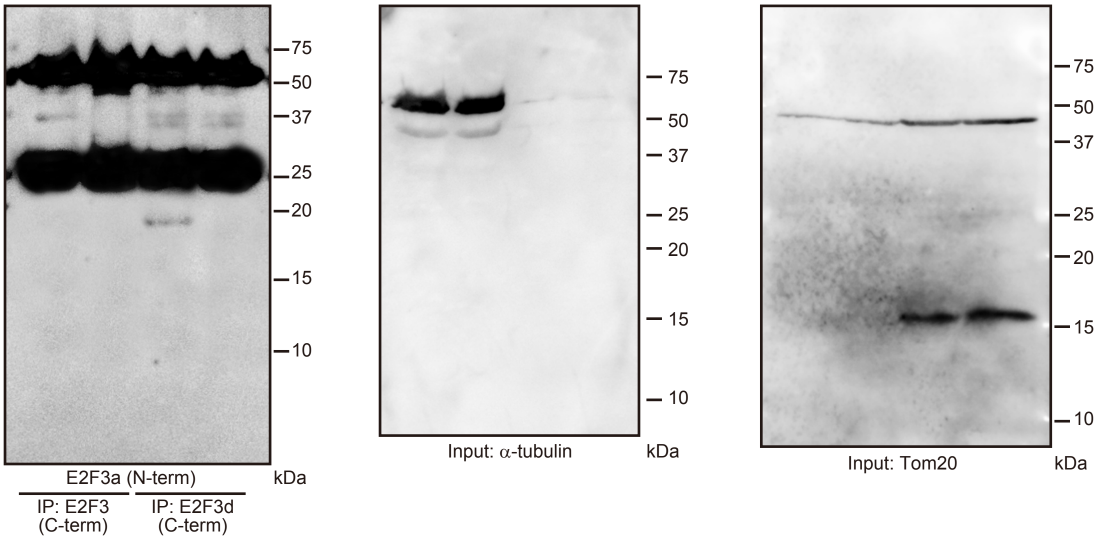

Supplement: Supplementary file 1 — Supplementary Information [file 42003_2018_246_MOESM1_ESM.pdf]
